# Efficient methods of isolation and purification of extracellular vesicles

**DOI:** 10.1186/s40580-025-00509-x

**Published:** 2025-09-25

**Authors:** Taewoon Kim, Jong Wook Hong, Luke P. Lee

**Affiliations:** 1 Department of Bionanotechnology, Graduate School, Hanyang University, Seoul, 04763 Korea; 2 Department of Medical and Digital Engineering, Graduate School, Hanyang University, Seoul, 04763 Korea; 3 Department of Bionanoengineering, Hanyang University, Gyeonggi-Do, 15588 Korea; 4 Harvard Medical School, Department of Medicine, Brigham and Women’s Hospital, Harvard University, Boston, MA 02115 USA; 5 Department of Bioengineering, University of California at Berkeley, Berkeley, CA 94720 USA; 6 Department of Electrical Engineering and Computer Science, University of California at Berkeley, Berkeley, CA 94720 USA; 7 Department of Biophysics, Institute of Quantum Biophysics, Sungkyunkwan University, Suwon, 16419 Korea; 8 Department of Chemistry & Nanoscience, Ewha Womans University, Seoul, 03760 Korea

**Keywords:** Extracellular vesicles, Exosome, Separation, Diagnostics, Therapeutics

## Abstract

Living cells produce nanometer scales of extracellular vesicles (EVs) that have attracted considerable interest due to their transformative effects on diagnostics and therapies for cancer and other diseases. While significant advancements have been made in grasping the physical and chemical foundations of separation techniques for EVs, challenges must be overcome to ensure effective EV purification for diverse life sciences and clinical applications. This review highlights the most significant developments in efficient isolation and purification methods for EVs in transformative medicine. We examine the basic structure of exosomes and how to obtain specimens containing exosomes and EVs from various body fluids. We investigate the principles of physical, chemical, and biological isolation methods of EVs. We systematically evaluate different designs of microfluidics-based EV purification methods. We provide a comprehensive overview of the applications of exosomes in the life sciences and medicine. The precise engineering of EV isolation and purification generates a high yield and purity, offering practical solutions for translational medicine.

**Graphical Abstract:**

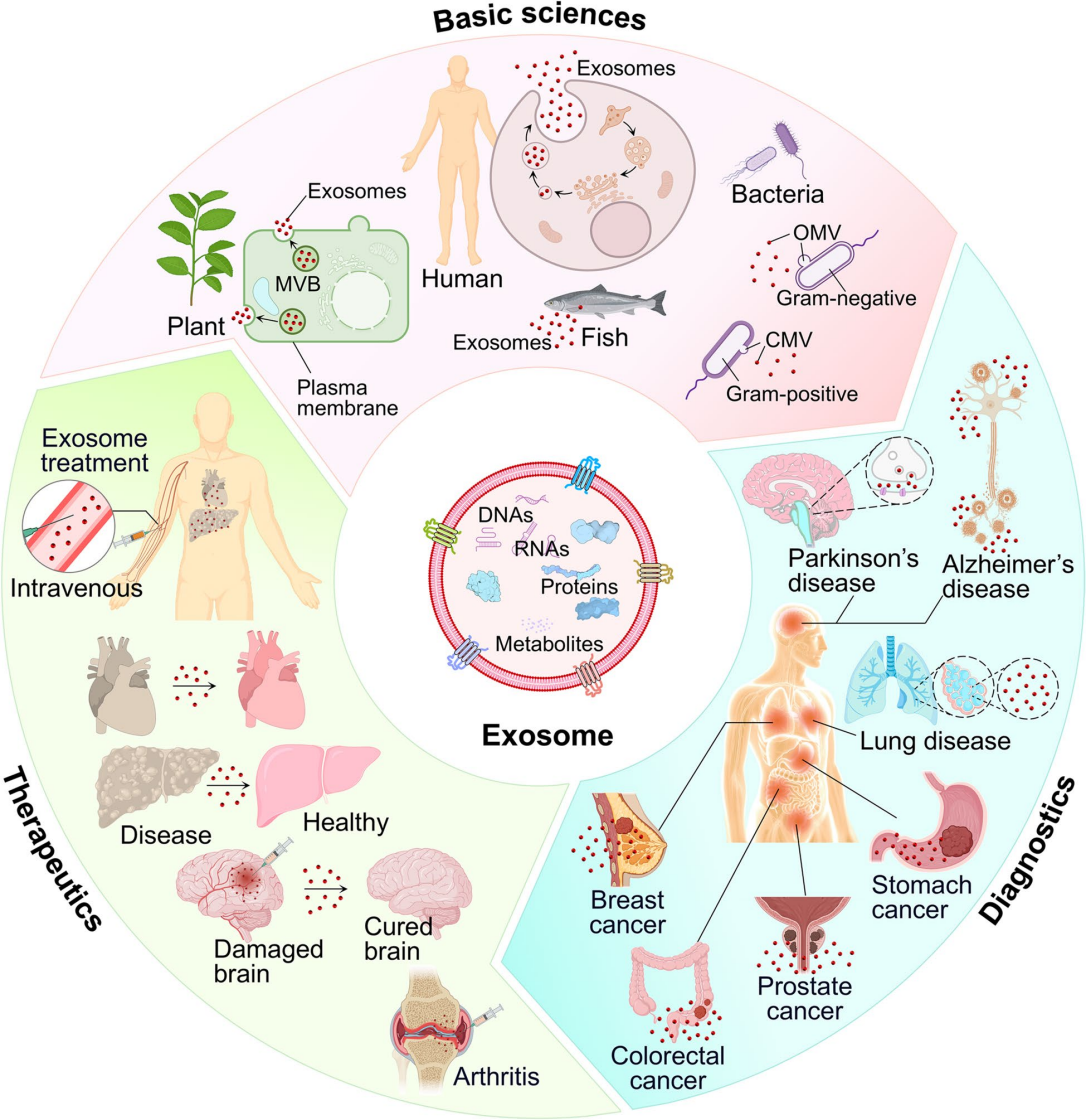

**Supplementary Information:**

The online version contains supplementary material available at 10.1186/s40580-025-00509-x.

## Introduction

Living cells produce nano-scale vesicles called extracellular vesicles (EVs), which are tiny particles released by cells into the surrounding environment, and they are classified into three main categories based on size and biological origin: exosomes, microvesicles, and apoptotic bodies [1].

Exosomes typically have a diameter of 30–200 nm [2,3] and are formed through the budding process of endosomal membranes [4,5]. This process leads to the formation of multivesicular bodies (MVBs) that fuse with the cell membrane, releasing exosomes into the extracellular space. Exosomes play a crucial role in regulating various cellular functions through interactions with other cells [6,7]. Microvesicles are larger than exosomes with a 100–1000 nm diameter and are formed through outward budding and fission of the plasma membrane. On the other hand, apoptotic bodies are generated during cell death and typically have a diameter of 50 nm to − 5 µm (BOX 1) [8–10].

**Box 1:**
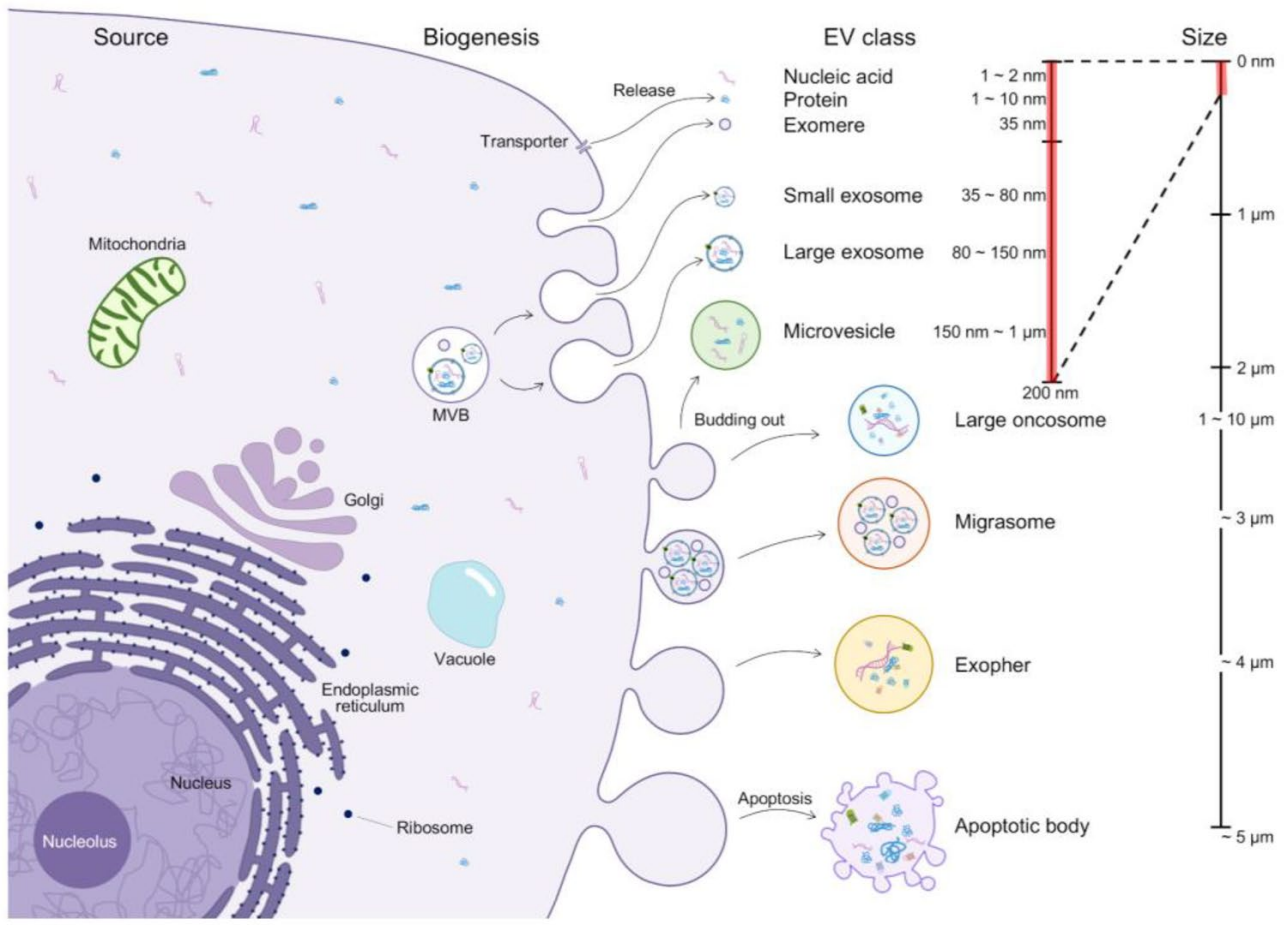
Schematic of nano/microvesicles that comprise the communication system of cells. Extracellular vesicles (EVs) containing exosomes are listed according to the size range. Evs include cargo substances of cells from the cytoplasm, mitochondria, golgi, plasma membranes, vesicles, and nuclei. Exosomes (diameter 35 to 150 nm) are produced through penetration of endosomes in cells to capture cytoplasmic components. After fusion of multivesicular bodies (MVBs) and plasma membranes, exosomes are released into extracellular space. Microvesicles are more heterogeneous in size (diameter 150 nm to 1 µm) and encapsulate cytoplasmic components through the budding and fission of cytoplasmic membranes. Apoptotic bodies (diameter 50 nm to 5 μm) are released according to the activation of the apoptosis pathway. Large oncosomes are abnormally large (diameter 1 to 10 μm), resulting from the discharge of membrane blends, and are associated with progressive diseases. Migrasome (diameter about 3 μm), which contains numerous small vesicles, grows at the intersection of the end of the contracting fiber. These fibers, which connect the vesicle and the primary cell, are eventually broken, and the vesicle is discharged into the extracellular space or absorbed directly by surrounding cells. Open nematodes and protein-secreting neurons can release neurotoxic proteins from large membrane-binding vesicles called exospheres. Evs can be characterized based on the expression of specific proteins associated with vesicle transport and biogenesis, such as tetraspanin and chromosomal proteins, maturation-related proteins, and thermal shock proteins. In addition, chromosomes or plasma membrane proteins have been proven to be abundant in Evs.

Since their discovery in 1983 during the maturation of red blood cells, exosomes have gained attention as a mechanism for removing intracellular proteins [11,12]. However, their biological importance and relevance to diseases were not fully understood at that time. It was not until 1987 when they were named exosomes by Professor Rose Johnstone [13] and subsequent research explored their role in cell-to-cell communication and disease [14–17]. Exosomes regulate cellular functions such as cell growth, division, and apoptosis by delivering bioactive molecules, including proteins, nucleic acids, and lipids, to target cells [18–21]. These bioactive molecules can originate from both the native cell and the extracellular environment [22], and they provide unique and complex cargo that reflects the cellular and physiological state of releasing cells [23,24]. Exosomes have been found in various body fluids such as blood [25], urine [26–28], saliva [29], sweat [30], tears [31], vaginal fluid [32], and cerebrospinal fluid [33], making them highly promising for diagnostic and therapeutic applications [34–39].

**Fig. 1:**
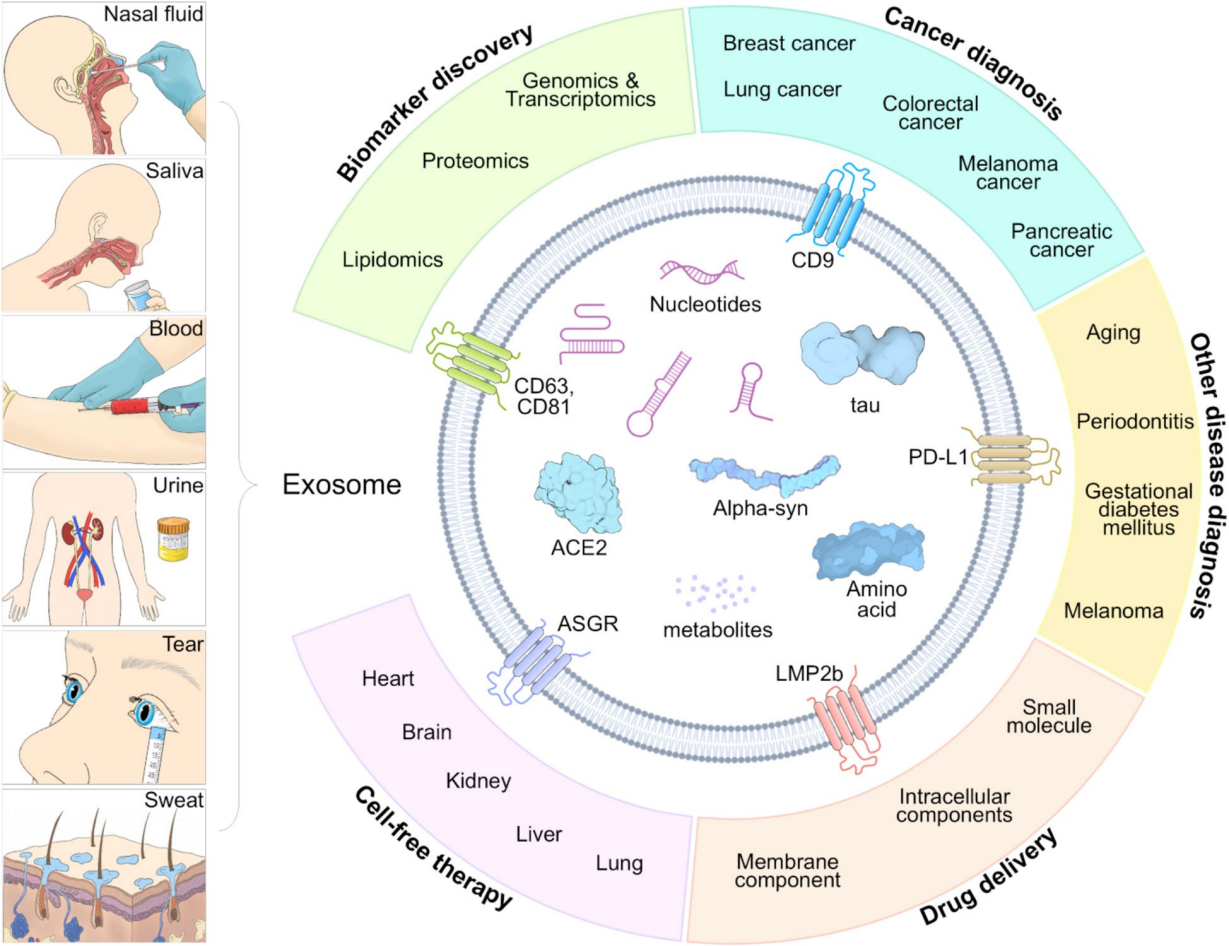
Body fluid-derived nanometer scale exosomes’ typical structure and application in diagnosis and therapy. Exosomes that can be separated from body fluids such as nasal fluid, saliva, blood, urine and sweat, etc., include various components of DNA, mRNA, miRNA, and protein. In the field of diagnosis, different cancers and diseases can be diagnosed through biomarker discovery. Exosomes have characteristics of cells of originations and are used for cell-free therapy and can be used for drug delivery through modifications

Exosomes hold great potential for novel applications in diagnosis, treatment, and medical research (Fig. 1) [40–42]. They encapsulate proteins, nucleic acids, and other molecules that can vary depending on specific conditions or diseases, making them valuable for disease diagnosis and prognostic prediction [43,44]. Using exosomes forms the basis for precision medicine through precise and early diagnosis [45–48]. Furthermore, exosomes play a role in tissue regeneration and repair, making them potential therapeutic agents [49]. For example, exosomes derived from stem cells are being investigated as possible tools [50–52] for treating neurodegenerative disorders such as Alzheimer's [53,54] and Parkinson's [55,56] diseases. Additionally, exosomes can be effectively employed as drug delivery systems [57,58]. They circulate stably within the body and can interact with specific receptors or ligands exposed on their surface, enabling selective delivery to specific tissues or cells. This suggests their potential as novel drug carriers for therapeutic applications [59,60].

However, physical damage or surface alterations of nanometer vesicles, including the disruption of the internal structure or surface proteins, can compromise the sensitivity of exosome diagnostics and limit their therapeutic applications [61]. To fully harness the potential of exosomes in medicine and biology, it is imperative to secure techniques for isolating exosomes without causing damage and ensuring reproducibility. Current exosome isolation methods have inherent limitations that can result in exosome damage, high variability in experimental outcomes, and hindered reproducibility [62]. Moreover, current methods face challenges in the large-scale production of undamaged exosomes, and the morphological and biological diversity of isolated exosomes pose difficulties in comparing results between experiments [63]. Therefore, there is a need to develop better methodologies that can isolate undamaged exosomes with reproducibility and enable large-scale production. Such advancements will maximize the potential of exosomes and contribute to revolutionary advancements in medicine and biology [64].

Various methods are used for exosome isolation, including ultracentrifugation [65], filtration [66], chromatography [67], polyethylene glycol (PEG) precipitation [68], and immunoaffinity capture [69]. Recently, with the advancements in nanotechnology and microfluidic technology, techniques such as inertial separation [70], field-flow fractionation (FFF) [71], acoustic separation [72], and pinched flow fractionation (PFF) [73–75] have been employed for exosome isolation. In particular, a tunable microfluidic system named Biologically intact Exosome Separation Technology (BEST) has been utilized for exosome isolation [27,28,76–79].

In this review, we extensively examine the advantages and limitations of different exosome isolation methods, comparing their efficiency, purity, and other factors using mathematical equations and calculations. By offering a comprehensive understanding of exosome isolation techniques, we aim to assist researchers in selecting the most reliable and efficient methods. This will contribute to substantial advancements in medicine and biology by using exosomes and extracellular vesicles (EVs).

## Separation of living nanoparticles, EVs, by traditional methods

### Ultracentrifugation

When a mixture is put in a centrifuge and rotated, the centrifugal force varies depending on the mass of the constituent material. The greater the mass of the constituent material, the greater the centrifugal force applied, and the material precipitates out [80,81]. The method to calculate the centrifugal force is as follows. $F=mr{w}^{2}$ Here, $F$, $m$, $r$, $w$ are centrifugal force, mass of matter, radius of circle, and angular velocity. Since the rotation radius of the material to be centrifuged varies depending on the rotor’s position, which is where the object to be centrifuged is placed, relative centrifugal force is mainly used considering the angular velocity and the rotation radius [82].

1 $$RCF=(1.118\times {10}^{-5}){(RPM)}^{2}r$$

Here, $RCF$, $RPM$, and $r$ are relative centrifugal force, revolutions per minute, and the radius of the rotating body, respectively. Relative centrifugal force is the ratio between gravity and centrifugal force acting on particles in a mixture, expressed in the form ‘*g*’ and denoted as ‘*x g*’ [83]. Therefore, the higher the RPM, the larger the mass, and the larger the radius, the higher the centrifugal force, which causes the heavy material to settle rapidly in the direction of the centrifugal force [84].

**Fig. 2:**
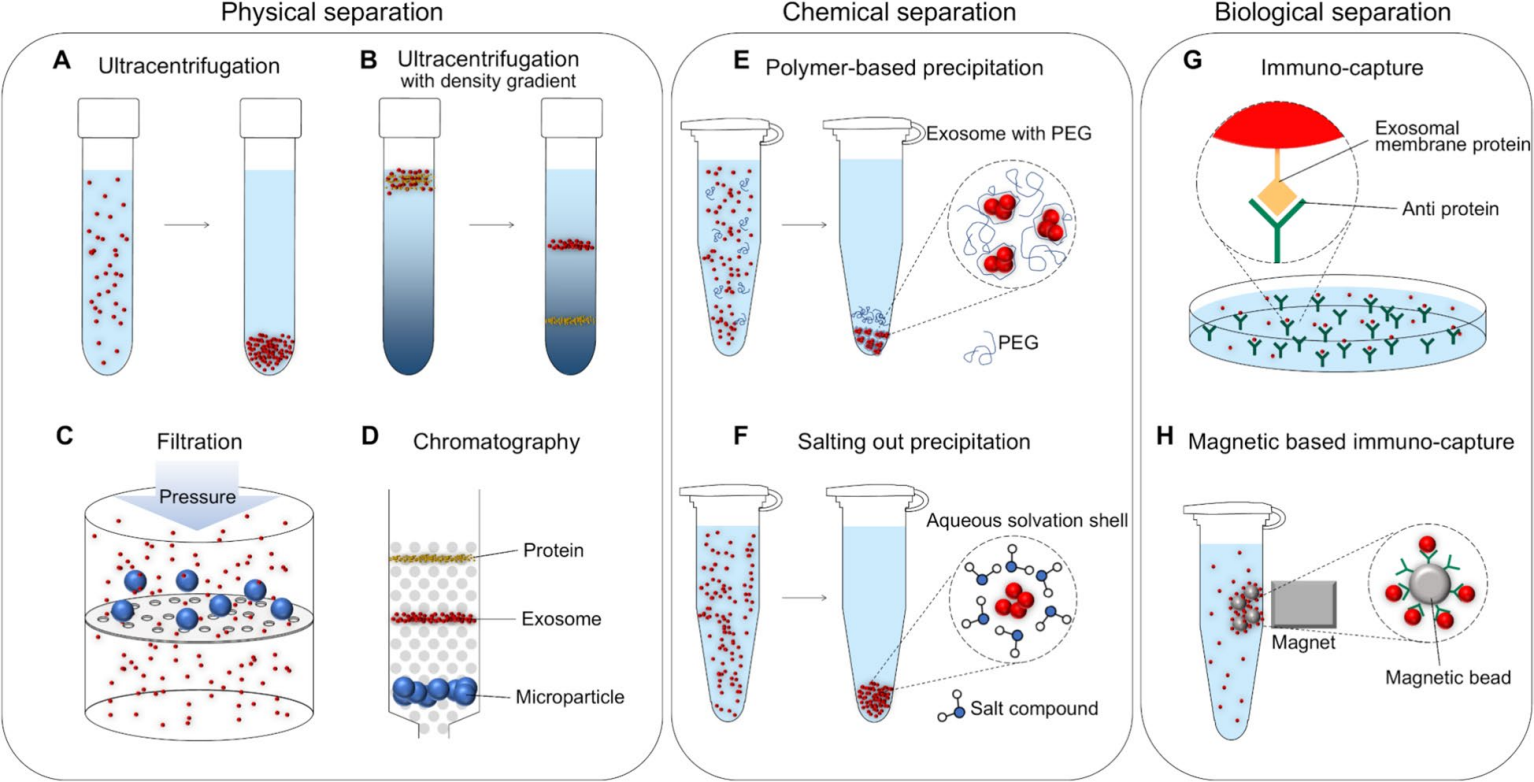
Principles of physical isolation, chemical precipitation, and immuno-capture-based living nanovesicle exosome separation techniques. **A** Ultracentrifuge that isolates exosomes using a robust centrifugal force generated at a high rotational speed. **B** Density gradient centrifugation using a buffer consisting of a density gradient so that the exosomes stop at the same point as the density of the exosomes. **C** Filtration by applying pressure with the sample to a filter with a pore size similar to exosomes. **D** Size exclusion chromatography utilizes different mobility of exosomes according to particle size through a porous structure. **E** Precipitation aggregates exosomes by forming a polyethylene glycol (PEG) network. **F** Salting out that precipitates exosomes by adding acetate to the buffer to adjust the pH. **G** Immuno-capture is based on the binding reaction between exosome membrane protein and anti-membrane proteins. **H** Magnetic immuno-capture that attaches antibodies to a magnetic bead to hold exosomes through an immune reaction and separate them by a magnetic field

Centrifugation can be divided into differential and density gradient centrifugation according to the separation method. Differential centrifugation is a method of sequentially settling and separating particles according to size and density through a series of centrifugal forces and duration (Fig. 2A and Supplementary Fig. 1A) [85]. This method is known as the gold standard method used for exosome isolation because it is economical in cost for consumables or reagents used for isolation and has excellent reproducibility in the process [86–88]. However, this method has a disadvantage in that large particles located at the top of the tube settle together with small particles at the bottom while settling [89]. In order to improve such co-precipitation, a method of resuspending the precipitate and centrifuging several times can be used, but in the repeated process of resuspending and centrifugation, the recovery rate of exosomes is lowered to 30%, and there is a possibility that exosomes may be damaged [90,91]. In addition, expensive equipment such as an ultracentrifuge is required to isolate the exosomes, and there is the inconvenience of undergoing centrifugation under various conditions. Therefore, developing a technology that can efficiently extract exosomes without expensive equipment is necessary.

As another method, density gradient centrifugation separates substances based on density differences using sucrose or cesium chloride (CsCl) solution, unlike differential centrifugation using a homogeneous solution (Fig. 2B and Supplementary Fig. 1B) [92–94]. The concentration of a molecular solute can be calculated using the following formula [95].

2 $$\frac{dC}{d\gamma }=-{\omega }^{2}MGQC(\gamma -{\gamma }_{0})$$

Here, *C*, γ, ω, *M*, *G*, and *Q* are the concentration of molecular solute, the function of position, the angular velocity of the centrifuge, the molecular weight of the molecule, given density gradient, and constants. Since the density of the solution in the tube increases as it goes down, mixing by convection during centrifugation can be prevented. In addition, since each material is distributed according to the height of the tube after centrifugation by the density gradient is completed, the density of the material can be calculated from the height of the tube [96]. Density gradient centrifugation is superior in separation efficiency and results in higher purity, and can prevent remixing of components separated by the density gradient of the solution. However, this method has disadvantages because preparing a concentration gradient solution before use is cumbersome and takes a long time to perform [97,98]. To address these limitations, ongoing research is focusing on simplifying gradient preparation through pre-formed or self-generating density media, as well as implementing automated fraction collectors coupled with real-time optical or sensor-based monitoring to identify exosome-rich layers more precisely.

### Filtration

Filtration is the simplest method to isolate exosomes from a sample through a membrane with a specific permeability without special equipment or harmful chemical reagents (Fig. 2C and Supplementary Fig. 1C) [99,100]. Filtration methods can be largely divided into dead-end filtration (DEF) and tangential flow filtration (TFF) [101]. DEF refers to a filtration process in which the flow direction of the fluid is the same as the filtration direction. First, filtration is performed using a filter membrane with a pore size of 0.8 µm or 0.45 µm to separate cells, cell debris, and large non-cellular vesicles from the sample [102]. After preliminary filtration, the exosomes are collected by passing through a filter membrane having a pore size of about 0.22 µm or 0.1 µm, considering the size of the exosomes [103,104]. Therefore, when separating exosomes using DEF, the pore size and material of the filtration membrane play an important role. High yield and purity of exosome isolation can be achieved by selecting a filtration membrane with a pore size similar to that of exosomes. In addition, DEF has the advantage of a relatively simple filtration process and rapid separation of exosomes from a large amount of samples [8]. However, there are disadvantages in that the problem of blocking the pores of the filtration membrane during the filtration process cannot be completely solved, and the exosomes may be damaged due to pressure changes generated during the filtration process [105]. In order to overcome these disadvantages, studies on isolating exosomes using other filtration techniques, such as TFF, are also being conducted. TFF is a filtration method in which the flow direction of the fluid is perpendicular to the filtration direction, which can effectively prevent clogging of the filtration membrane and improve the stability of the equipment [106]. However, when the filtration method alone is used, the isolated exosomes can be significantly contaminated with floating proteins outside the exosomes, such as albumin [107]. In order to solve this problem, new methods are needed to use filtration in combination with other exosome separation techniques, such as ultracentrifugation and chromatography, or to prevent or minimize the binding in the filtration process.

Recently, Fei's Exodus method was introduced to isolate exosomes without clogging [31,108]. This technique uses acoustic fluid streaming to reduce the fouling effect of double membrane filters, improving throughput, yield, and purity. A comprehensive overview of filtration-based exosome isolation can be read here.

3 $${q}_{L}=\frac{{k}_{m}}{\mu }\frac{\partial {P}_{L2}}{\partial x}$$

Here, ${q}_{L}$, ${k}_{m}$, ${P}_{L2}$, μ, and *x* are liquid velocity, the permeability of the filter medium, the pressure of the liquid in the filter medium, fluid viscosity, and absolute space coordinate [109].

### Size-exclusion chromatography, SEC

Size exclusion chromatography is a method for separating solutes based on their molecular size [81]. It was developed in the 1950s to study the elution characteristics of low and high molecular weight carbohydrates and proteins [110,111]. SEC utilizes a gel filtration system where a stationary phase composed of silica or polymer particles forms a mesh structure with uniform micro-sized pores through which molecules can diffuse (Fig. 2D and Supplementary Fig. 1D) [112]. These pores are small enough to exclude large molecules but not small enough to exclude small molecules. As a result, larger molecules cannot pass through the pores and are rapidly eluted, while smaller molecules enter the pores and undergo a slower filtration process [92]. This principle is applied to separate larger vesicles first, followed by the elution of exosomes. Additionally, SEC can improve the purity of exosomes by removing impurities such as unwanted proteins or lipids from the sample [113]. This is essential to exosome analysis, as impurities can hinder accurate analysis and reliable results.

However, SEC requires a long separation time to distinguish slight differences between components within a column to achieve high resolution and sufficient separation [114,115]. This limits the amount of sample that can be processed at once, making it inefficient for handling large sample volumes, often requiring multiple analyses [116,117]. In addition, SEC has limitations in discriminating between exosomes and microvesicles of the same size [107,118,119], necessitating combination with immunocapture methods for specific surface marker-based separation [67].

Recent developments have sought to overcome these limitations. Robinson et al. demonstrated the clinical applicability of SEC for 1 mL plasma by directly comparing it to alternative methods, validating its reproducibility and purity for diagnostic workflows [120]. More notably, Kapoor et al. introduced a size-exclusion fast protein liquid chromatography (SE-FPLC) approach that operates under high pressure with optimized flow rates and high-density gel-packed columns [121]. This innovation enables EV recovery in under 20 min with ~ 88% yield, effectively addressing the slow speed and low throughput of traditional SEC while preserving purity. These advancements point toward SEC becoming a more practical and scalable option for both research and clinical applications.

4 $${K}_{SEC}=\frac{t-{t}_{excl}}{{t}_{perm}-{t}_{excl}}$$

Here, ${K}_{SEC}$, $t$, ${t}_{excl}$, and ${t}_{perm}$ are partition coefficient, retention time, excluded particle retention time, and permeable particle retention time [122].

### Polymer-based precipitation

The polymer-based precipitation method is a separation method based on the principle of precipitating exosomes in biological fluids using polymers (Fig. 2E and Supplementary Fig. 2A) [123,124]. One of the common polymers used for polymer-based exosome isolation is polyethylene glycol (PEG) [125], aqueous PEG envelops dozens or hundreds of exosomes together to form exosome aggregates that can be readily precipitated by low-speed centrifugation [68]. Representative commercial products include Total Exosome Isolation Reagent (Invitrogen, United States) [126] or ExoQuick (System Biosciences, United States) [90]. They need to precipitate the exosomes using a specific polymer and separate the desired exosomes from the precipitate containing the exosomes.

The polymers used in the polymer-based precipitation method are relatively inexpensive and do not require additional expensive equipment or consumables [127,128]. In addition, it can be easily performed according to a simple procedure, and a large number of samples can be processed in parallel, saving time and effort. However, the polymer-based precipitation method needs to be fixed with the purity of the isolated exosomes. In particular, there are many cases in which non-exosomal vesicles and other solutes co-precipitate, and an additional purification process after separation may be required [129]. In addition, there is a disadvantage in that other endoplasmic reticulum of similar size, such as exosomes and microvesicles, cannot be distinguished [80]. To overcome these limitations, we are focusing on developing polymers that better match the properties of exosomes by tailoring the composition of the polymers [130].

Another approach is to combine polymer-based precipitation with other separation techniques [131]. For example, techniques such as size exclusion chromatography or immunoprecipitation can discriminate between exosomes and microvesicles and can be used as an additional purification step after polymer-based precipitation. After all, the polymer-based precipitation method itself is not a perfect exosome isolation method, but it is widely used because of its simplicity and efficiency, and its limitations can be overcome through combination with other techniques. Future research is expected to optimize these combinations and develop more efficient and pure exosome isolation methods.

### Salting out precipitation

Salting out precipitation is a method of precipitating exosomes in solution by neutralizing the surface charge of exosomes with acetate using the property that the surface of exosomes is negatively charged (Fig. 2F) [62,132,133]. The precipitation depends on the pH and the salt concentration and optimized precipitation occurs when the pH is 4.75 with 0.1 M acetate [134]. The protocol is as follows. The biological fluid sample is centrifuged to remove cells, debris, and large vesicles (500*g* for 30 min, 12,000*g* for 30 min). Then 0.1 volume of sodium acetate buffer (1.0 M pH 4.75) was mixed with supernatants, kept on ice for 30–60 min, and incubated at 37 °C for 5 min. Exosomes are precipitated by centrifugation (5000*g* for 10 min). After washing the pellet with 0.1 M sodium acetate buffer, centrifugation is performed under the same conditions to resuspend the pellet in HBS (HEPES buffered saline). If necessary, additional precipitation procedures could be repeated (Supplementary Fig. 2B) [135]. This procedure is convenient, technically simple, and saves time without the need for expensive equipment [136]. However, these precipitation methods ultimately cannot resolve particle heterogeneity and are not specific for exosomes or other EVs. Thus, these methods could lead to the isolation of non-exosomal particles in addition to exosomes, potentially leading to erroneous findings and flawed conclusions. Future research should focus on refining the physicochemical parameters, such as salt type, concentration, and pH, to selectively neutralize exosomal surface charges while minimizing co-precipitation of contaminants.

5 $${V}_{rel,UP}=\frac{{V}_{UP}}{{V}_{i}}$$

Here, ${V}_{rel,UP}$, ${V}_{UP}$, and ${V}_{i}$ are relative volume of the upper phase, the volume of the upper phase, and volume of the corresponding binary system water without salt [137].

### Immunoaffinity capture

Exosome isolation using immunoaffinity is a highly specific technique, and exosomes are isolated using antibodies against specific surface proteins (Fig. 2G and Supplementary Fig. 2C) [138]. This method has the advantage of obtaining high-purity exosomes compared to other isolation techniques. Various proteins and receptors, such as CD9, CD63, and CD81, exist on the membrane of exosomes, and these are used as antibodies in immunoaffinity separation [139]. These antibodies are immobilized on a plate [140], a microfluidic device [141], or the most commonly using magnetic beads [142,143] (Fig. 2H and Supplementary Fig. 2D). Through this method, only exosomes having a specific protein binding to the antibody can be isolated, and impurities can be removed.

Immunoaffinity-based isolation of exosomes has several significant limitations. One of the main limitations is selectivity, as specific markers may not be present or recognized in all exosomes [144]. This means that the separation efficiency can be low due to the limited specificity of the markers. Additionally, the process of immunoaffinity capture involves the immobilization of antibodies on magnetic beads to recognize exosomes, followed by the release of exosomes from the antibodies using a magnetic field. The binding process between exosomes and antibodies requires specific temperature conditions (4 °C) and a minimum incubation time (1 h or more), and the release of exosomes requires an appropriate buffer with a specific salt concentration (NaCl 1 M, pH 7.0, 200 μL) [145]. These requirements contribute to the high cost of immunoaffinity-based techniques, making them generally suitable for specific research purposes or small-scale sample applications [146].

Nevertheless, exosome isolation using immunoaffinity can be helpful in studies targeting specific proteins [147]. Through this method, it is possible to isolate and analyze specific subsets of exosomes that are expressed in specific disease states. In particular, it can be essential in disease diagnosis using exosomes. However, it should be noted that this method can only be applied when known exosomal surface proteins and corresponding antibodies are available, which is an inherent limitation.

6 $$q= \frac{{Q}_{max}*c}{{K}_{d}+c}$$

Here, ${Q}_{max}$ is the equilibrium binding capacity, $q$ and c represent the stationary and mobile phase protein concentrations, and ${K}_{d}$ is the equilibrium dissociation constant [148].

## Separation of living nanoparticles by nanoliter scale fluidic devices

### Inertial separation

**Fig. 3:**
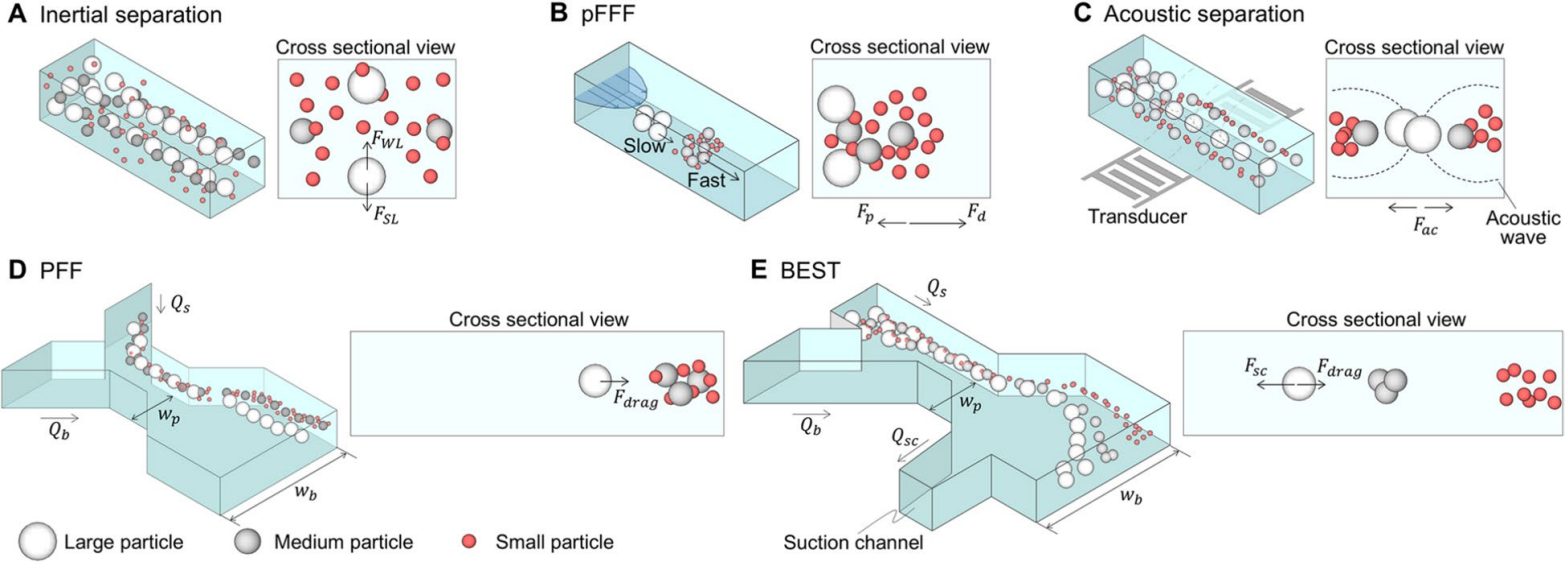
Nanoliter scale fluidic controls and microfluidic separation techniques and their conditions for exosome separation. **A** Inertial separation uses the difference responses and separates them in the concentrating location of the particle according to the difference in inertial forces. **B** Pressure field flow fractionation (pFFF) that sorts of particles by external force and then separates particles according to different diffusion distances. **C** Acoustic separation that separates the fluid into a node or anti-node according to the particle characteristics by giving a constant vibration to the fluid. **D** Pinched flow fractionation (PFF), which sorts particles on the wall and separates them according to the difference in center of gravity. **E** Biologically intact exosome separation technology (BEST) amplifies the difference in the center of gravity of particles by controlling the flow rate in different fluid geometry. ${F}_{WL}$: wall-induced lift force, ${F}_{SL}$: shear gradient lift force, ${F}_{P}$: force by pressure, ${F}_{d}$: force by diffusion, ${F}_{ac}$: force by an acoustic wave, ${F}_{drag}$: drag force, ${F}_{sc}$: force by suction flow. ${Q}_{s}$: sample flow rate, ${Q}_{b}$: buffer flow rate, ${Q}_{sc}$: suction flow rate, ${w}_{p}$: width of pinched segment, ${w}_{b}$: width of broadened segment

Inertial microfluidics or nanofluidics is a technique that utilizes the inertial forces generated at the microscale to separate particles [149]. In this method, a specific flow velocity is induced in a small channel through which fluid flows, and particles are introduced into the fluid. Due to the diverse sizes and shapes of particles, they interact with the fluid flow and move either toward the channel walls or the center [150]. This separation phenomenon relies on two main fluidic forces, as illustrated in Fig. 3A [151,152]. Firstly, the shear gradient lift force pushes particles toward the channel walls as they flow through the microchannel. Secondly, the wall-induced lift force, resulting from the interaction between particles and the channel walls, moves particles toward the center axis of the channel. As a result, larger particles shift towards the wall-affiliated equilibrium position, while smaller particles move closer to the center axis equilibrium position [153].

Inertial microfluidics separates biological particles, including blood cells and exosomes [154], and offers high throughput due to fast fluid flow [155]. Additionally, it can separate particles without needing specific markers or fluorescent tags [156]. However, to maximize separation efficiency in inertial microfluidics, optimization of device design and operating conditions is required, and additional preprocessing may be necessary for complex samples like blood cells [157].

Recent innovations have integrated inertial separation principles into multifunctional microfluidic platforms. For instance, Zhao et al. reported a fully integrated centrifugal microfluidic system that combines rapid exosome isolation via inertial and centrifugal forces with on-chip glycan profiling for point-of-care diagnostics [158]. This approach enables high-purity isolation in under 30 min and directly couples separation with downstream biochemical analysis, thereby minimizing sample loss and processing time. Such integration illustrates the potential of inertial microfluidics not only for high-throughput EV separation but also for embedding analytical modules to deliver complete diagnostic workflows on a single platform.

### Field-flow fractionation, FFF

Field-flow fractionation (FFF) is a separation technique that utilizes the interplay between crossflow and opposing diffusion forces to move particles and separate them based on their size [159]. Unlike other methods, FFF doesn't rely on filters or stationary phases. Instead, it allows particles to elute from different positions depending on their size, making it a highly efficient and versatile technique [160,161].

**Fig 4:**
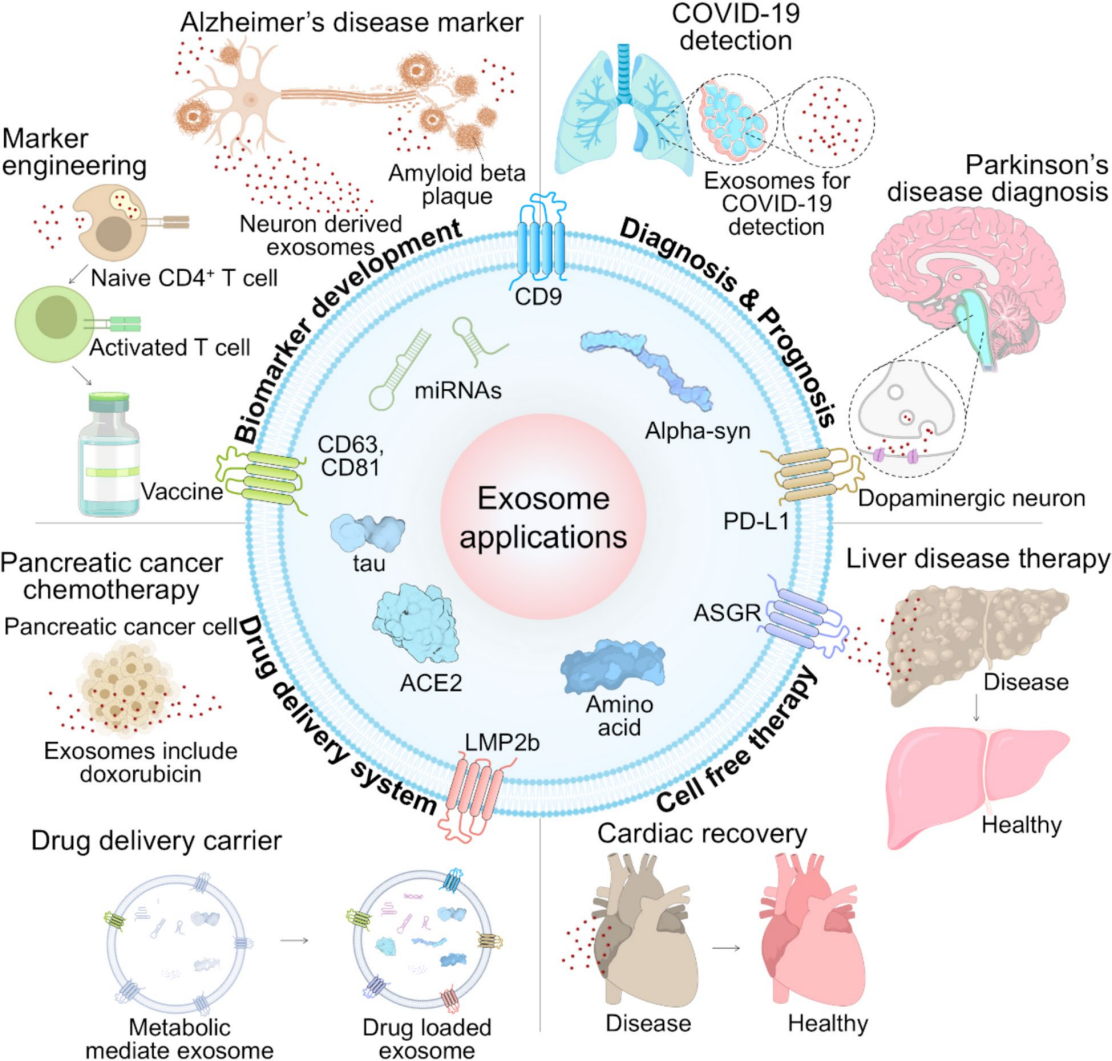
Schematics of exosome applications in biology and medicine. Exosomes are applied in various fields such as biomarker development, diagnosis of diseases including COVID-19, Parkinson’s disease, pathological application, cell-free therapy, and drug delivery system

As shown in Fig. 4B, particles are driven by the interplay of crossflow and opposing diffusion forces, forming an exponential steady-state layer near the lower (accumulative) semipermeable membrane. The concentration relative to that at the membrane surface is described by the expression $\mathrm{exp}(-\frac{x}{l})$, where $x$ is the distance above the wall and $l$ is the characteristic thickness of the layer. The parameter $l$ is given by $l=\frac{D}{U}$, where $D$ is the diffusion coefficient and $U$ is the sideways velocity imposed by the crossflow. It is helpful to express $l$ in the dimensionless form, $\uplambda =\frac{l}{w}$, where $w$ is the channel width spacing between membranes. We have $\uplambda =\frac{D}{{U}_{w}}$. If lateral velocity, $U$, is replaced by volumetric crossflow, ${V}_{C}$, over the channel (membrane) area (breadth $\mathrm{a}$ times length $\mathrm{L}$), the equation becomes $\uplambda =\frac{D{V}^{0}}{{V}_{c}{w}^{2}}$, where ${V}^{0}=awL$ is the column void volume [162].

One of the key advantages of FFF is its gentle separation approach. It allows particles to move through a fluid medium rather than forming a pellet or passing through a narrow pore. This means that the original buffer of the sample can be used for elution, preserving its integrity. FFF is widely used for extracting and separating exosomes, tiny vesicles involved in cell communication. By adjusting parameters such as crossflow velocity, membrane properties, and sample volume, researchers can efficiently and precisely separate exosomes from complex biological samples.

### Acoustic separation

Acoustic separation using microfluidics is a technique that leverages acoustic forces to manipulate and separate particles within microscale fluidic systems [163,164]. This approach combines the advantages of acoustics and microfluidics to achieve precise and efficient particle separation.

In acoustic separation, sound waves are generated and applied to a fluidic channel or chamber, creating acoustic radiation forces (Fig. 3C) [165]. These forces exert pressure variations on the particles suspended in the fluid, leading to their manipulation and segregation based on size, density, or other physical properties. Depending on the specific configuration and operational parameters, the acoustic forces in acoustic separation can manifest as radiation force, acoustic streaming, or acoustic standing waves. In the case of standing waves, they induce periodic pressure fluctuations within the liquid present in the microchannel. These fluctuations generate an acoustic radiation force (${F}_{R}$) that pushes particles toward the pressure nodes:

7 $${F}_{R}=-\left(\frac{\pi {{{\beta }_{f}V}_{p}p}_{0}^{2}}{2\lambda }\right)\varnothing \left(\beta ,\rho \right)\mathrm{sin}\left(\frac{4\pi x}{\lambda }\right)$$

In Equation, ${\beta }_{f}$, ${V}_{p}$, ${p}_{0}$, $\varnothing$, $\beta$, $\rho$, $\lambda$, and $x$ are compressibility of fluid, volume of particle, acoustic pressure, acoustic contrast factor, compressibility, density, wavelength of the acoustic waves, and distance from a pressure node, respectively.

Acoustic separation techniques in microfluidics have demonstrated remarkable capabilities in various applications [166,167]. These techniques have successfully isolated and enriched rare cells, including circulating tumor cells, stem cells, and immune cells, from complex biological samples. Acoustic separation also finds utility in the sorting and purifying of EVs, including exosomes, which play critical roles in intercellular communication and disease biomarker discovery. Recently, Naquin et al. introduced the Acoustic Separation and Concentration of Exosomes for Nucleotide Detection (ASCENDx) platform, which integrates acoustic separation with downstream molecular analysis in a continuous-flow microfluidic format [168]. This system not only isolates exosomes with high purity and recovery but also concentrates them directly in a detection-ready state, enabling rapid nucleic acid assays without intermediate purification steps.

### Pinched flow fractionation, PFF

Pinched Flow Fractionation, first proposed by Yamada et al., is a microfluidic technology that separates micrometer-sized particles according to size using the microchannel structure, consisting of a pinched and broadened segment [73]. The PFF microchannel features two inlets and is structured such that the channels from these inlets converge at the pinched segment. This pinched segment is then connected to the broadened segment, where the channel width is wider than in the pinched segment, enabling the separation of particles (Fig. 3D).

The detailed separation principle of PFF is as follows: Fluids containing the particles are to be separated, and particle-free fluids are simultaneously introduced through each inlet, meeting in the pinched segment [169]. Here, the widths of the two fluids in the pinched segment are determined based on the flow rates of each fluid. When the flow rate of the particle-free fluid is higher than that of the particle-containing fluid, the width of the particle-containing fluid narrows while the width of the particle-free fluid widens. When the width of the particle-containing fluid becomes smaller than the radius of the particles within the fluid, the particles align along one side wall [170,171].

Notably, as particles transition from the narrow pinched segment to the broadened segment while being aligned based on their radii, the width of the broadened segment expands, and the fluid flow transforms radially [172]. Furthermore, due to the drag force, the distance between particles with different radii expands based on the ratio of the widths between the pinched and broadened segments [173,174]. This increased distance between particles according to their radii enhances the separation of particles, particularly in the broadened segment, providing clearer size-based separation compared to the pinched segment [175]. For a particle with a radius $r$, its position ${L}_{B}$ within the broadened segment, measured from the upper wall, is determined by the aspect ratio between the pinched and broadened segments and can be expressed as

8 $${L}_{B}\equiv \frac{{w}_{b}}{{w}_{p}}\times r$$

where ${w}_{b}$ is the width of the broadened segment, and ${w}_{p}$ is the width of the pinch segment.

According to the aforementioned principles, PFF provides a higher separation resolution, characterized by results in larger differences in width between the pinched and broadened segments. This technology can separate biological samples such as red and white blood cells [176]. However, it's important to note that this technique has limitations, mainly in its application to micro-sized particles. Recent study show that integrating it with other microfluidic methods, such as inertial focusing, deterministic lateral displacement, or active field-assisted separation, can improve precision and extend applicability to nanoscale particles [177]. Such hybrid systems combine PFF’s high-resolution focusing with secondary forces to fine-tune trajectories, enabling efficient isolation of extracellular vesicles from complex fluids and positioning PFF as a promising next-generation nano-bio separation platform.

### Biologically intact exosome separation technology, BEST

In BEST (Fig. 3E), a chip is used where solutions containing particles and buffer are injected separately through different inlets. These solutions travel at various flow rates until they reach a pinched segment. Here, the particles are pushed towards a side wall and constrained to follow streamlines determined by their respective center of mass positions, which vary according to size. Afterward, the solution enters a broadened segment of the device where the streamlines and the particles they contain are separated and collected through different outlets. An additional side channel can induce an asymmetric flow distribution and further enhance the separation effect in the broadened segment. Shin et al. [76] reported using a PFF device with nine outlets and a tunable magnification side channel, the suction channel, for size-based separation of extracellular vesicles (EVs). The allocation of particles within the broadened segment and outlets was finely controlled by adjusting the flow rates of the two inlets and modulating the withdrawing flow of the suction channel. The efficiency of the device was demonstrated by its successful separation of exosomes and apoptotic bodies from cell culture media [76,77,178].

A simple model was developed to describe the BEST system. This model was based on the linear Stokes equation, incorporating the boundary conditions for flow velocity and particle alignment in the expansion segment. The distance from the upper wall of the broadened segment to the center of mass of the particles is defined by the formula:

9 $${L}_{B}=\frac{{w}_{b}}{{w}_{p}}(\frac{{Q}_{s}+{Q}_{b}}{{Q}_{s}+{Q}_{b}-{Q}_{sc}})r$$

Here, ${w}_{b}$, ${w}_{p}$, ${Q}_{s}$, ${Q}_{b}$, ${Q}_{sc}$, and $r$ are the width of broadened segment, the width of the pinch segment, the flow rate of sample, the flow rate of buffer, the flow rate of suction, and the radius of particle. The ability to separate particles and vesicles, ranging from nanoscale to microscale, directly from biological samples like blood and urine without prior treatments holds immense promise for advancing the fields of biological pharmaceuticals and diagnostics.

While BEST has demonstrated high-resolution separation of extracellular vesicles without prior sample pretreatment, further optimization is needed to enhance throughput for large clinical sample volumes and to integrate downstream analytical modules for point-of-care diagnostics. Combining BEST with complementary techniques such as immunoaffinity capture or real-time optical detection could improve specificity and enable multiplex biomarker analysis. Advances in microfabrication, flow control, and automation are expected to facilitate the development of fully integrated BEST-based platforms, paving the way for scalable, reproducible, and clinically validated EV isolation systems applicable to both therapeutic manufacturing and precision diagnostics.

## Ideal method for nanometer scale exosome isolation and purification

Since each isolation technique mentioned above has advantages and disadvantages (Table 1) [179–181], only some universally accepted methods are considered ideal for isolating exosomes in the EV field. Researchers understand that more than these techniques alone can provide optimal results, and therefore, it is widely recognized that a combined approach using multiple techniques may yield the best outcomes.

**Table 1:** Summary of exosome isolation methods with their advantages and disadvantages

Method	Advantage	Disadvantage
Physical separation		
Ultracentrifugation	- Gold standard, widely used - No need for expensive reagents	- Multiple steps & consequent variations - Co-precipitation of large particles - Physical damages of exosomes, time-consuming - Requires expensive ultracentrifuge - Multiple steps
Ultracentrifugation with density gradient	- Higher purity than differential UC - Prevents remixing during separation	- Long preparation and operation time - Labor-intensive - Requires preparation of gradient solutions - Multiple steps
Filtration	- Simple and rapid - No harmful reagents - Handle some extended volumes	- Filter clogging - Exosome deformation from pressure - Protein contamination - Multiple steps
Size exclusion chromatography	- Preserves exosome structure - Removes proteins/lipids effectively	- Poor discrimination between exosomes and similar-sized microvesicles - Long separation time - Limited sample volume per run
Chemical separation		
Polymer-based precipitation	- Relatively inexpensive - Handle multiple samples in parallel	- Low purity, co-precipitation of contaminants - Cannot distinguish EV subtypes - Require further purification after the process - Multiple steps
Salting-out	- Relatively inexpensive - Fast precipitation without expensive instruments	- Low specificity for exosomes - Co-isolation of non-EV particles - Multiple steps
Biological separation		
Immuno-capture	- High specificity and purity - Enables isolation of EV subpopulations	- Only for existing marker-dependent - Miss EVs over the capture capability - High cost - Long incubation time, complex buffer requirements - Multiple steps
Magnetic capture	- High selectivity using antibodies or aptamers on magnetic beads - Easy separation via magnetic field - Compatible with small sample volumes	- Rely heavily on known surface markers - Potential loss during elution - Limited scalability for extended volume - Multiple steps
Fluidic base separation		
Field flow fractionation, FFF	- Single step & continuous separation	- Intrinsic limitation of purity - Low throughput for large volumes - Requires specialized equipment
Acoustic separation	- High purity - Single step & continuous separation	- Potential damage by external force - Requires precise acoustic control - Low throughput for large volumes - Need throughput increase
Biologically intact Exosome Separation Technology, BEST	- High yield & high purity - Single step & continuous separation - Handle from 100 µL of sample - Direct processing of cell culture, blood, urine, etc. - Damage-free and contamination-free intact EVs	- Need throughput increase

Table 2 summarizes various methods for isolating exosomes of approximately 30 to 200 nm and other particles smaller than animal cells and bacteria. Specifically, yield and purity for each method were calculated and compared based on published data [27,28,69,73,76–79,83,91,94,95,100,109,113,122,133,137,143,148–150,161,162,164,165] regarding exosome isolation. We define yield as the number of exosomes in the sample after separation compared to the number before separation, Yield [%] = (Number of exosomes after separation / Number of exosomes before separation) × 100, through modification of previous reports on exosome recovery [108,182] (Supplementary Fig. 3). Isolation of exosomes based on ultracentrifugation can cause disruption or deformation of exosomes and results in lower yield [90,183,184]. Therefore, a high yield indicates minimal exosome loss during the separation process. Exosome production per milliliter refers to the number of exosomes ultimately isolated from a unit volume of 1 ml of sample. We also define purity as the proportion of exosomes in the final isolated sample, Purity [%] = (Number of exosomes / Total number of particles in the isolated sample) × 100 (Supplementary Fig. 3). This concept of purity is an advanced one based on direct exosome counting and differs from the purity of exosomes based on indirect postulation with the protein quantity of exosomes [185–187]. In order to communicate effectively and ensure robust, precise quantification, as well as high-quality production of EVs, it is essential to standardize the separation and purification of exosomes according to the actual quantity of particles.

**Table 2:** Quantitative comparison of exosomes from various samples and exosome derivatives from different separation techniques

Separation method	Equation	Yield of exosome [%]	Particles or weight per ml	Target	Sample	Labeling	Exosome separation	Purity of exosome [%]	Continuous separation	Ref
Physical separation										
Ultracentrifugation	$RCF=1.1{(RPM)}^{2}{10}^{-5}r$	~ 7	~ 4.8 × 10^10^	Exosome	Serum		○	~ 77	^X^	[83,91]
Ultracentrifugation with density gradient	$\frac{dC}{d\gamma }=-{\omega }^{2}MGQC\left(\gamma -{\gamma }_{0}\right)$	n.a	~ 2.1 × 10^11^	Exosome	Cell culture		○	n.a	^X^	[94,95]
Filtration	${q}_{l}=\frac{{k}_{m}}{\mu }\frac{\partial {P}_{l2}}{\partial x}$	n.a	~ 1.8 × 10^10^	Exosome	Urine		○	n.a	^X^	[100,109]
Size exclusion chromatography	${K}_{SEC}=\frac{t-{t}_{e}}{{t}_{p}-{t}_{e}}$	~ 6	~ 9.0 × 10^9^	Exosome	Serum		○	~ 48	^X^	[91,122]
Chemical separation										
Polymer-based precipitation	n.a	~ 41	~ 7.3 × 10^10^	Exosome	Serum		○	~ 52	^X^	^[[91]]^
Salting-out	${V}_{r, u}=\frac{{V}_{u}}{{V}_{i}}$	n.a	~ 3.3 × 10^11^	Exosomal Protein	Cell culture		○	n.a	^X^	[133,137]
Biological separation										
Immuno-capture	$q= \frac{{Q}_{max}*c}{{K}_{d}+c}$	n.a	n.a	Exosomal Protein	Plasma	Required	○	n.a	^X^	[69,148]
Magnetic capture	$R=\frac{{[P]}_{mb}}{{[P]}_{pl}}$	n.a	~ 4.8 μg	Exosomal Protein	Cell culture	Required	○	n.a	^X^	[113,143]
Fluidic base separation										
Inertial separation	$L= \frac{3\pi \mu {D}_{h}^{2}}{4\rho {U}_{f}{a}^{3}}(\frac{H}{{C}_{l}^{-}}+\frac{W}{{C}_{l}^{+}})$	n.a	n.a	Microparticle	Polystyrene		^X^	n.a	○	[149,150]
Field flow fractionation, FFF	$l=\frac{D}{\left|U\right|}$	n.a	~ 1.7 × 10^9^	Exosome	Serum		○	~ 76	○	[161,162]
Acoustic separation	${F}_{r}=-\left(\frac{\pi {{{\beta }_{f}V}_{p}p}_{0}^{2}}{2\lambda }\right)\varnothing \left(\beta ,\rho \right)\mathrm{sin}\left(\frac{4\pi x}{\lambda }\right)$	~ 82	~ 8.4 × 10^10^	Exosome	Whole blood		○	~ 98	○	[164,165]
Pinched flow fractionation, PFF	${L}_{b}=\frac{{w}_{b}}{{w}_{p}}\times r$	n.a	~ 5.0 × 10^5^	Microparticle	Polystyrene		^X^	n.a	○	[73]
Biologically intact Exosome Separation Technology, BEST	${L}_{b}=\frac{{w}_{b}}{{w}_{p}}(\frac{{Q}_{s}+{Q}_{b}}{{Q}_{s}+{Q}_{b}-{Q}_{m}})r$	~ 99	~ 1.8 × 10^12^	Exosome	Cell culture Vaginal discharge		○	~ 97	○	[27,28,76–79]

($RCF$: relative centrifugal force, $RPM$: revolutions per minute,$r$: distance of the particles from the center of rotation, $C$: concentration of molecular solute, $\gamma$: function of position, $\omega$: angular velocity of the centrifuge, $M$: molecular weight of the molecule, $G$: given density gradient, $Q$: constants, ${q}_{l}$: liquid velocity, ${k}_{m}$: permeability of the filter medium, ${P}_{l2}$: pressure of liquid in the filter medium, $\mu$: liquid viscosity, $x$: absolute space coordinate, ${K}_{SEC}$: partition coefficient, $t$: retention time, ${t}_{e}$: retention time of excluded particle, ${t}_{p}$: retention time of permeable particle, ${V}_{r,u}$: relative volume of the upper phase, ${V}_{u}$: volume of the upper phase, ${V}_{i}$: volume of the corresponding binary system water without salt, ${Q}_{max}$: equilibrium binding capacity, $q$: stationary phase protein concentrations, $c$: mobile phase protein concentrations, ${K}_{d}$: equilibrium dissociation constant, $R$: capture rate, ${[P]}_{mb}$: concentration of protein captured on magnetic beads, ${[P]}_{pl}$: concentration of protein in plasma, $L$: equilibrium position; ${C}_{l}$: nondimensional lift coefficient; $\rho$: fluid density; $W$: longer channel dimension; $H$: shorter channel dimension; $a$: particle diameter; ${D}_{h}$: channel of hydraulic diameter; ${U}_{f}$: average flow velocity; $\mu$: coefficient of friction, $l$: characteristic elevation of the particle cloud; $D$: diffusion coefficient; $U$: field-induced velocity, ${F}_{r}$: acoustic radiation force; ${\beta }_{f}$: compressibility of fluid; ${V}_{p}$: volume of particle; ${p}_{0}$: acoustic pressure; $\varnothing$: acoustic contrast factor; $\beta$: compressibility; $\rho$: density; $\lambda$: wavelength of the acoustic waves; $x$: distance from a pressure node, ${L}_{b}$: the distance of particles from the upper broadened channel wall; ${w}_{p}$: width of the pinched segments; $r$: radius of particle; ${w}_{b}$: the width of the broadened channel; ${Q}_{b}$: Flow rate of buffer; ${Q}_{m}$: Flow rate of magnification)

We have confirmed that yields of biologically intact exosome separation technology, acoustic separation, and polymer-based precipitation range from 98.7 to 41.4%. Relatively higher yields of biologically active separation and acoustic separation are attributed to the precise control of extremely small fluid volumes, on the order of tens of nanoliters, without the need for harsh external forces that can cause physical damage or deformation [1–3]. Fluidic-based separation of exosomes is versatile to high-throughput systems through design modifications of chips and parallelization. In contrast, non-fluidic-based separation techniques such as ultracentrifugation and PEG precipitation deal with larger sample volumes at once but have clear limitations of yield and reproducibility [4] due to physical stress from strong centrifugal forces [5] and operator-dependent variability [6] in multi-step processes [1,7,8]. Purity of obtained exosomes through acoustic separation, biologically-active exosome separation and ultracentrifugation was 98.4%, 96.8%, and 76.6%, respectively. PEG or polymer-based precipitation methods often co-isolate non-exosomal components such as microvesicles and apoptotic bodies and provide exosomes with structural damages or chemical contaminations [9].

Differential ultracentrifugation has been associated with potential drawbacks in exosome isolation, including potential damage to exosomes and alterations in their proteome, lipidome, and/or genome [90]. Filtration, on the other hand, can result in the deformation and breakup of larger vesicles due to pressure and contact with filter membranes [105]. Additionally, PEG-based methods can lead to the co-precipitation of non-exosomal components and modification of exosomal protein signatures [118]. However, certain nanoliter scale fluidic approaches, such as acoustic and flow amplification techniques, have emerged as promising alternatives to overcome or mitigate these limitations. These methods offer minimally invasive, rapid, and high-purity isolation of exosomes. This is particularly advantageous in isolating exosomal fractions from blood plasma, where low-density lipoproteins can mimic exosomes and interfere with subsequent analysis if using differential ultracentrifugation. Moreover, microfluidic-based platforms bypass many procedural and mechanical drawbacks of conventional approaches by enabling single-step, automated operation with reduced sample handling, minimal shear stress, and significantly lower sample volume requirements. These improvements not only streamline workflows but also enhance reproducibility and scalability, providing a practical toward clinical-grade EV isolation. However, because most current fluidic approaches exhibit relatively low processing speeds for samples, large volumes takes a relatively long time. To shorten the separation time, development such as parallel type, high-speed implantation, or increasing the size of the chip itself is necessary.

A comparison of each microfluidic system is shown in Fig. 4. Throughput, purity, and yield were compared for each separable particle size. As a result of the comparison, acoustic and BEST methods could separate nanoparticles, and other technologies could separate microparticles. As discussed above, these individual exosome isolation techniques have their limitations. Therefore, to achieve the highest yield and purity, a combination of multiple isolation methods, such as differential ultracentrifugation, filtration, or PEG-based retrieval, can be employed. By integrating different technologies, it becomes possible to obtain intact and highly purified exosomes. Combining various methods compensates for the limitations of each technique and allows for more effective exosome isolation.

## Conclusions

We have assessed the current isolation or purification methods of living nanoparticles called EVs. Considering the varied origins of exosomes, it is crucial to develop an optimized isolation technique that minimizes uncertainties and inconsistencies in exosomal research. Researchers must balance achieving high purity and efficiency in isolating exosomes while considering the specific downstream applications of the vesicles. Finding a method that effectively addresses these factors is essential to ensure reliable and meaningful results in exosome studies. A combined optimized protocol is advisable to systematically evaluate differential ultracentrifugation, filtration, PEG-based precipitation, immunoaffinity capture, inertial separation, FFF, Acoustic separation, PFF, and BEST. The data obtained from trustworthy samples can significantly accelerate the discovery of new diagnostic biomarkers and therapeutic applications involving exosomes. Regardless of the selected methodology, we strongly advise researchers to validate the exosome isolation technique before experimenting, particularly when working with novel biofluids or samples. This validation step is crucial for ensuring the reliability and reproducibility of the experimental outcomes and enhancing the overall quality of exosome-related research. In comparison with classical reviews on nanoparticle-based exosome detection, which have largely focused on general biological aspects or qualitative overviews of isolation methods, our review synthesizes new knowledge by integrating both traditional and emerging micro/nanofluidic techniques into a unified framework. We further incorporate precise quantitative models for yield and purity, and present the mathematical formulations describing the underlying separation principles, enabling readers to connect fundamental principles with practical workflows for optimized EV isolation. A technology with an intact exosome isolation method still needs to be developed. Acoustic separation could also be used in particle concentration through fine manipulation of particles, which can be a breakthrough in field areas that require high-sensitivity signals, such as electrical and optical diagnostic devices. BEST could be developed as a stable and straightforward exosome separation method if the separation is accelerated or mass-produced. It could be utilized in unresolved biological phenomena, such as intercellular signaling, pharmacological responses, and mechanisms underlying cancer cell metastasis (Fig. 4).

## Supplementary Information

**Additional file 1.**

## Data Availability

All data are available in the main text or the supporting information.
