# Correction: Efficient methods of isolation and purification of extracellular vesicles

**DOI:** 10.1186/s40580-025-00523-z

**Published:** 2025-12-26

**Authors:** Taewoon Kim, Jong Wook Hong, Luke P. Lee

**Affiliations:** 1 Department of Bionanotechnology, Graduate School, Hanyang University, Seoul, 04763 Korea; 2 Department of Medical and Digital Engineering, Graduate School, Hanyang University, Seoul, 04763 Korea; 3 Department of Bionanoengineering, Hanyang University, Gyeonggi‑Do, 15588 Korea; 4 Department of Medicine, Brigham and Women’s Hospital, Harvard Medical School, Harvard University, Boston, MA 02115 USA; 5 Department of Bioengineering, University of California at Berkeley, Berkeley, CA 94720 USA; 6 Department of Electrical Engineering and Computer Science, University of California at Berkeley, Berkeley, CA 94720 USA; 7 Department of Biophysics, Institute of Quantum Biophysics, Sungkyunkwan University, Suwon, 16419 Korea; 8 Department of Chemistry & Nanoscience, Ewha Womans University, Seoul, 03760 Korea

**Correction to: Nano Convergence (2025) 12: 45**

10.1186/s40580-025-00509-x

In this article [1], Box 1, Figures 1, 2, 3, 4 and Table 1 appeared incorrectly and have now been corrected in the original publication. The correct version of Box 1, Figs. 1, 2, 3, 4 and Table 1 are presented with this correction article. The original article has been updated.

**Box 1:**
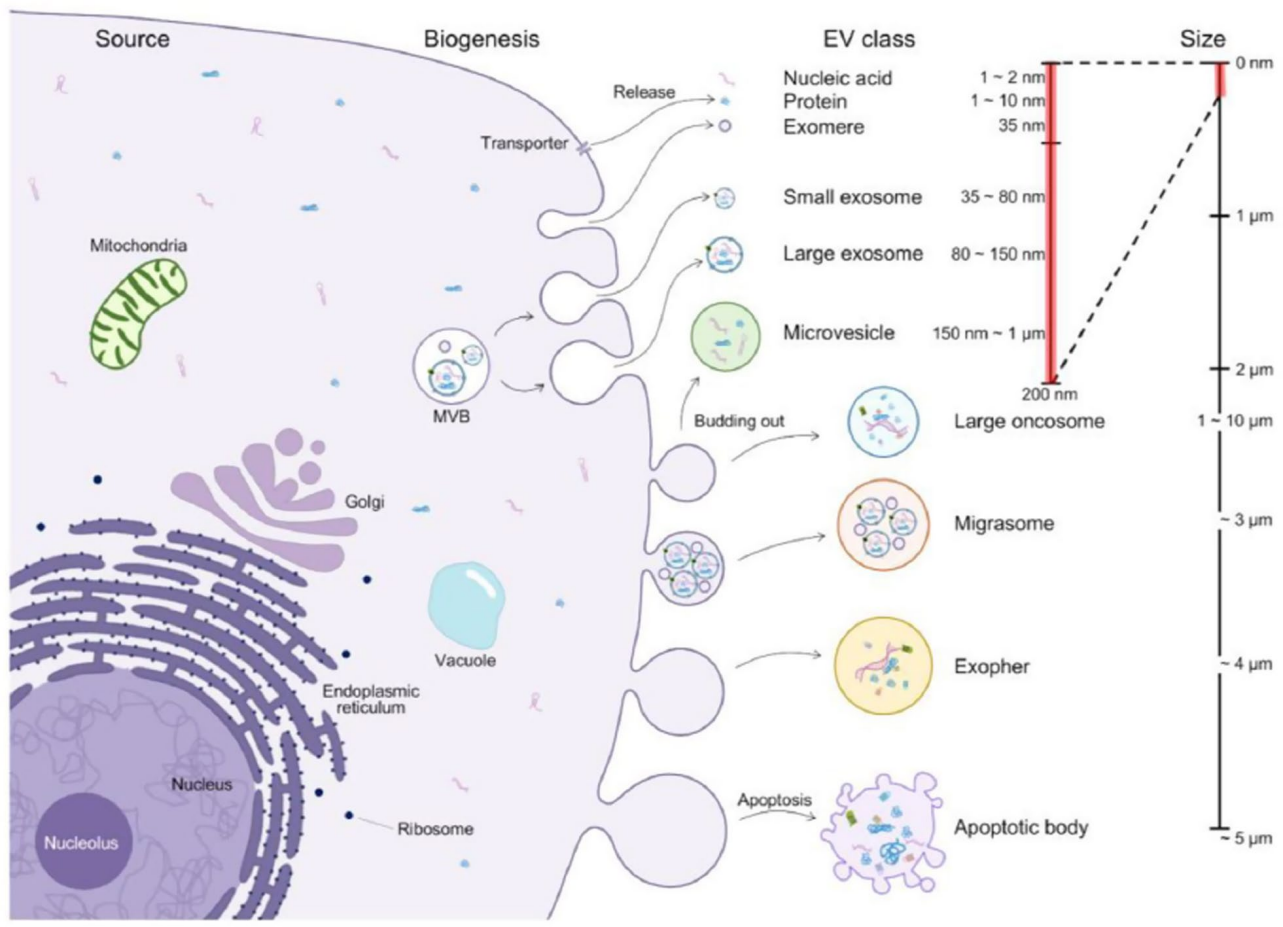
Schematic of nano/microvesicles that comprise the communication system of cells. Extracellular vesicles (EVs) containing exosomes are listed according to the size range. Evs include cargo substances of cells from the cytoplasm, mitochondria, golgi, plasma membranes, vesicles, and nuclei. Exosomes (diameter 35 to 150 nm) are produced through penetration of endosomes in cells to capture cytoplasmic components. After fusion of multivesicular bodies (MVBs) and plasma membranes, exosomes are released into extracellular space. Microvesicles are more heterogeneous in size (diameter 150 nm to 1 μm) and encapsulate cytoplasmic components through the budding and fission of cytoplasmic membranes. Apoptotic bodies (diameter 50 nm to 5 μm) are released according to the activation of the apoptosis pathway. Large oncosomes are abnormally large (diameter 1 to 10 μm), resulting from the discharge of membrane blends, and are associated with progressive diseases. Migrasome (diameter about 3 μm), which contains numerous small vesicles, grows at the intersection of the end of the contracting fiber. These fibers, which connect the vesicle and the primary cell, are eventually broken, and the vesicle is discharged into the extracellular space or absorbed directly by surrounding cells. Open nematodes and protein-secreting neurons can release neurotoxic proteins from large membrane-binding vesicles called exospheres. Evs can be characterized based on the expression of specific proteins associated with vesicle transport and biogenesis, such as tetraspanin and chromosomal proteins, maturation-related proteins, and thermal shock proteins. In addition, chromosomes or plasma membrane proteins have been proven to be abundant in Evs

**Fig. 1:**
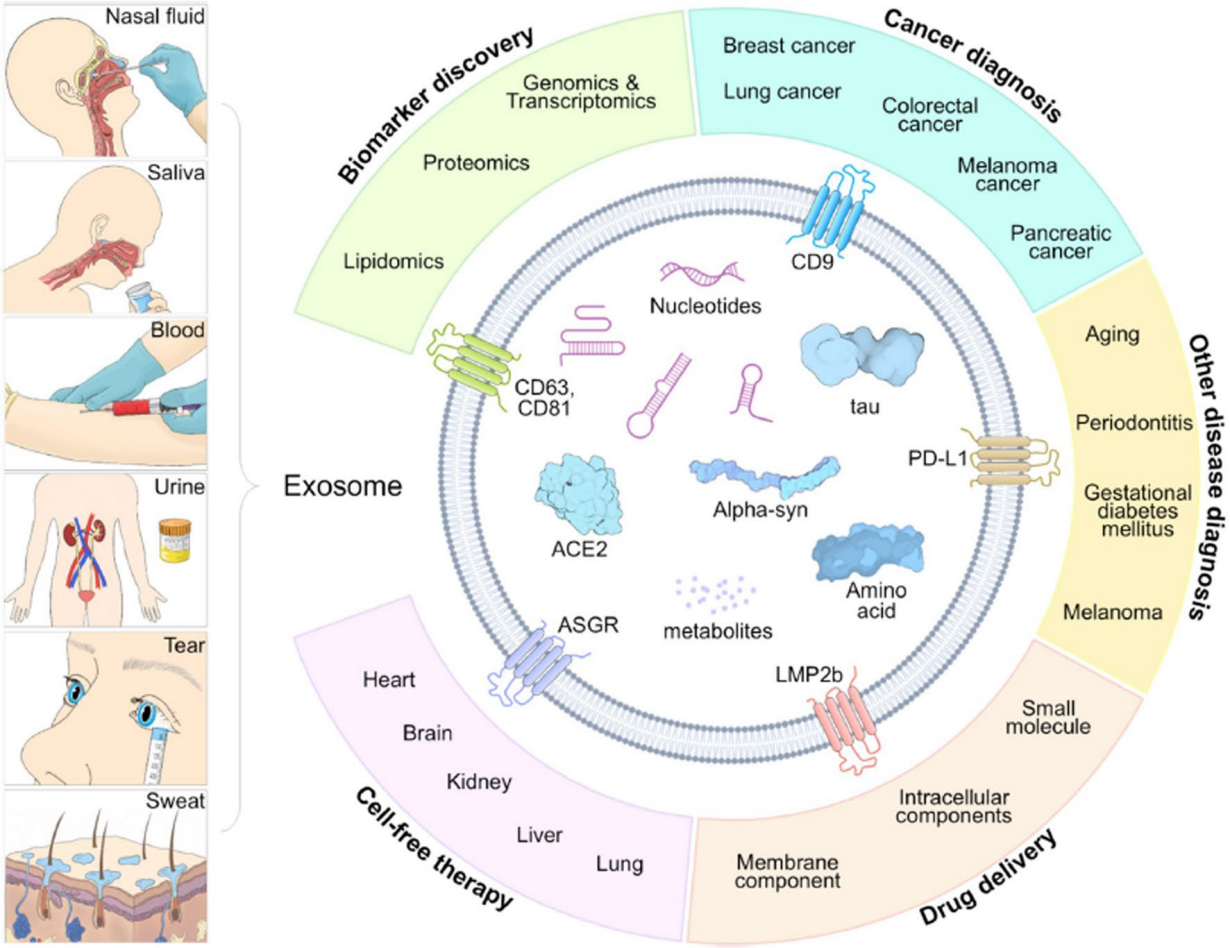
Body fluid-derived nanometer scale exosomes’ typical structure and application in diagnosis and therapy. Exosomes that can be separated from body fluids such as nasal fluid, saliva, blood, urine and sweat, etc., include various components of DNA, mRNA, miRNA, and protein. In the field of diagnosis, different cancers and diseases can be diagnosed through biomarker discovery. Exosomes have characteristics of cells of originations and are used for cell-free therapy and can be used for drug delivery through modifications

**Fig. 2:**
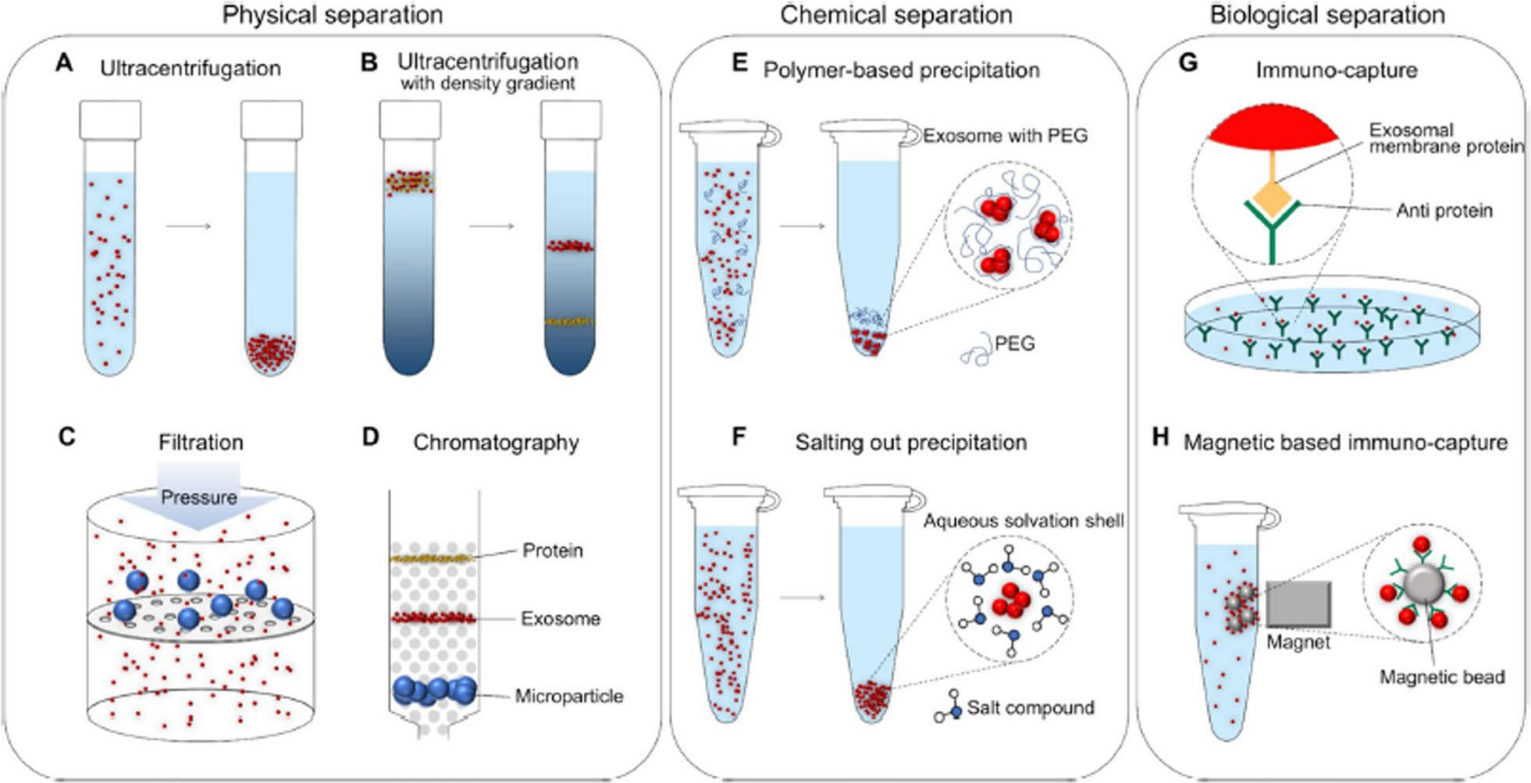
Principles of physical isolation, chemical precipitation, and immuno-capture-based living nanovesicle exosome separation techniques. **A** Ultracentrifuge that isolates exosomes using a robust centrifugal force generated at a high rotational speed. **B** Density gradient centrifugation using a buffer consisting of a density gradient so that the exosomes stop at the same point as the density of the exosomes. **C** Filtration by applying pressure with the sample to a filter with a pore size similar to exosomes. **D** Size exclusion chromatography utilizes different mobility of exosomes according to particle size through a porous structure. **E** Precipitation aggregates exosomes by forming a polyethylene glycol (PEG) network. **F** Salting out that precipitates exosomes by adding acetate to the buffer to adjust the pH. **G** Immuno-capture is based on the binding reaction between exosome membrane protein and anti-membrane proteins. H Magnetic immuno-capture that attaches antibodies to a magnetic bead to hold exosomes through an immune reaction and separate them by a magnetic field

**Fig. 3:**
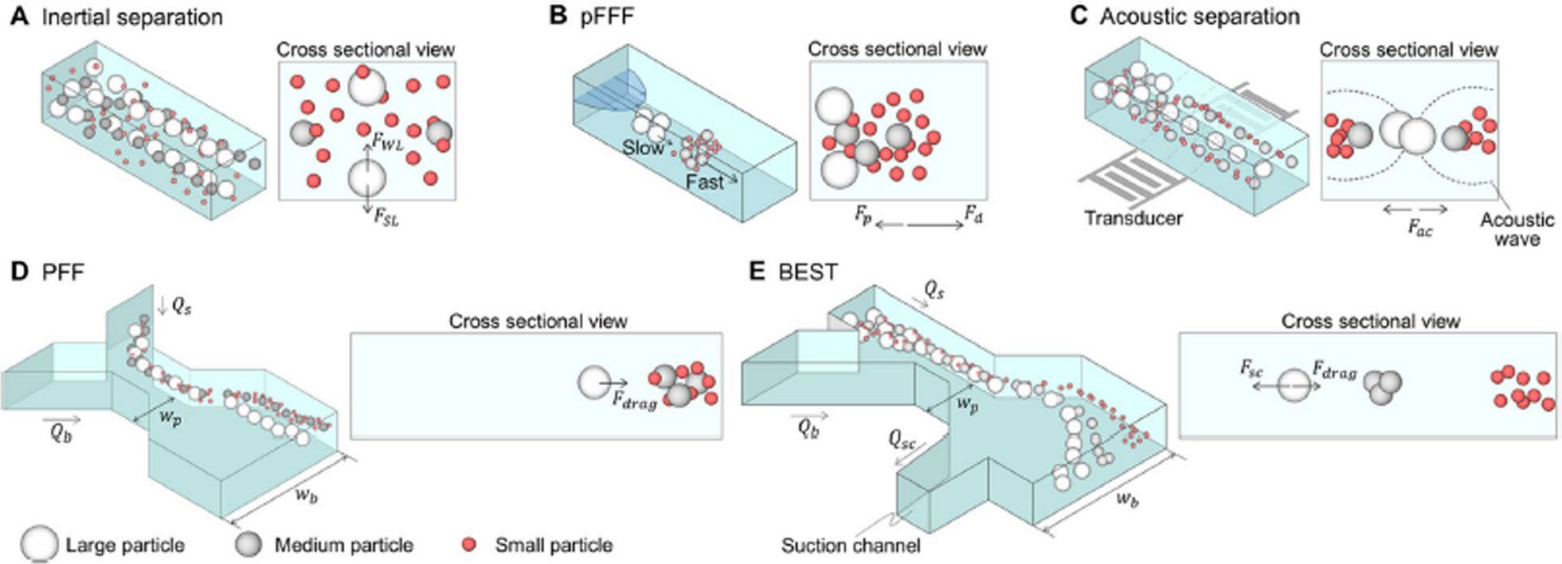
Nanoliter scale fluidic controls and microfluidic separation techniques and their conditions for exosome separation. **A** Inertial separation uses the difference responses and separates them in the concentrating location of the particle according to the difference in inertial forces. **B** Pressure field flow fractionation (pFFF) that sorts of particles by external force and then separates particles according to different diffusion distances. **C** Acoustic separation that separates the fluid into a node or anti-node according to the particle characteristics by giving a constant vibration to the fluid. **D** Pinched flow fractionation (PFF), which sorts particles on the wall and separates them according to the difference in center of gravity. **E** Biologically intact exosome separation technology (BEST) amplifies the difference in the center of gravity of particles by controlling the flow rate in different fluid geometry. FWL: wall-induced lift force, FSL: shear gradient lift force, FP: force by pressure, Fd: force by diffusion, Fac: force by an acoustic wave, Fdrag: drag force, Fsc: force by suction flow. Qs: sample flow rate, Qb: buffer flow rate, Qsc: suction flow rate, wp: width of pinched segment, wb: width of broadened segment

**Fig. 4:**
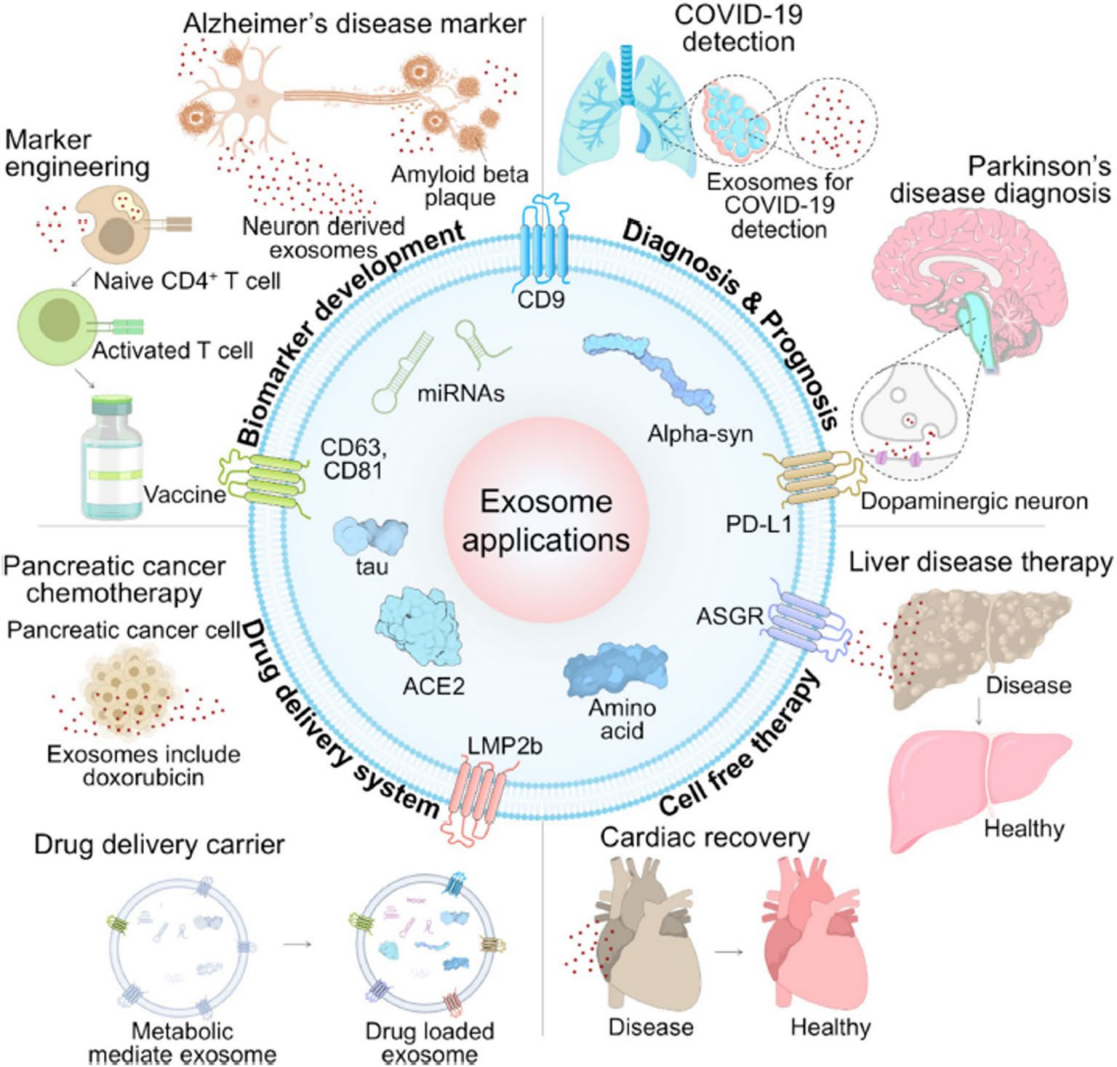
Schematics of exosome applications in biology and medicine. Exosomes are applied in various fields such as biomarker development, diagnosis of diseases including COVID-19, Parkinson’s disease, pathological application, cell-free therapy, and drug delivery system

**Table 1:** Summary of exosome isolation methods with their advantages and disadvantages

Method	Advantage	Disadvantage
Physical separation		
Ultracentrifugation	- Gold standard, widely used - No need for expensive reagents	- Multiple steps & consequent variations - Co-precipitation of large particles - Physical damages of exosome, time-consuming - Requires expensive ultracentrifuge - Multiple steps
Ultracentrifugation with density gradient	- Higher purity than differential UC - Prevents remixing during separation	- Long preparation and operation time - Labor-intensive - Requires preparation of gradient solutions - Multiple steps
Filtration	- Simple and rapid - No harmful reagents - Handle some extended volumes	- Filter clogging - Exosome deformation from pressure - Protein contamination - Multiple steps
Size exclusion chromatography	- Preserves exosome structure - Removes proteins/lipids effectively	- Poor discrimination between exosomes and similar-sized microvesicles - Long separation time - Limited sample volume per run
Chemical separation		
Polymer-based precipitation	- Relatively inexpensive - Handle multiple samples in parallel	- Low purity, co-precipitation of contaminants - Cannot distinguish EV subtypes - Require further purification after the process - Multiple steps
Salting-out	- Relatively inexpensive - Fast precipitation without expensive instruments	- Low specificity for exosomes - Co-isolation of non-EV particles - Multiple steps
Biological separation		
Immuno-capture	- High specificity and purity - Enables isolation of EV subpopulations	- Only for existing marker-dependent - Miss EVs over the capture capability - High cost - Long incubation time, complex buffer requirements - Multiple steps
Magnetic capture	- High selectivity using antibodies or aptamers on magnetic beads - Easy separation via magnetic field - Compatible with small sample volumes	- Rely heavily on known surface markers - Potential loss during elution - Limited scalability for extended volume - Multiple steps
Fluidic base separation		
Field flow fractionation, FFF	- Single step & continuous separation	- Intrinsic limitation of purity - Low throughput for large volumes - Requires specialized equipment
Acoustic separation	- High purity - Single step & continuous separation	- Potential damage by external force - Requires precise acoustic control - Low throughput for large volumes - Need throughput increase
Biologically intact Exosome Separation Technology, BEST	- High yield & high purity - Single step & continuous separation - Handle from 100 µL of sample - Direct processing of cell culture, blood, urine, etc - Damage-free and contamination-free intact EVs	- Need throughput increase
