# Supplementary material for: Efficient methods of isolation and purification of extracellular vesicles
Source: Nano Converg. 2025 Sep 25;12:45. doi: 10.1186/s40580-025-00509-x (PMC12463815; doi:10.1186/s40580-025-00509-x)
Supplement: Supplementary file 1 — Additional file 1. [file 40580_2025_509_MOESM1_ESM.docx]

**Supplementary Information**

**Efficient Methods of Isolation and Purification of Extracellular Vesicles**

Taewoon Kim^1^, Jong Wook Hong^1,2,3*^ and Luke P. Lee^4-8*^

^1^Department of Bionanotechnology, Graduate School, Hanyang University, Seoul 04763, Korea.

^2^Department of Medical and Digital Engineering, Graduate School, Hanyang University, Seoul 04763, Korea.

^3^Department of Bionanoengineering, Hanyang University, Gyeonggi-do 15588, Korea.

^4^Harvard Medical School, Harvard University; Department of Medicine, Brigham and Women’s Hospital, Boston, Massachusetts, USA.

^5^Department of Bioengineering, University of California at Berkeley, Berkeley, CA, USA.

^6^Department of Electrical Engineering and Computer Science, University of California at Berkeley, Berkeley, CA, USA.

^7^Department of Biophysics, Institute of Quantum Biophysics, Sungkyunkwan University, Suwon, Korea.

^8^Department of Chemistry & Nanoscience, Ewha Womans University, Seoul, Korea.

*Corresponding author: jwh@hanyang.ac.kr (J.W. Hong); lplee@bwh.harvard.edu (L. P. Lee)


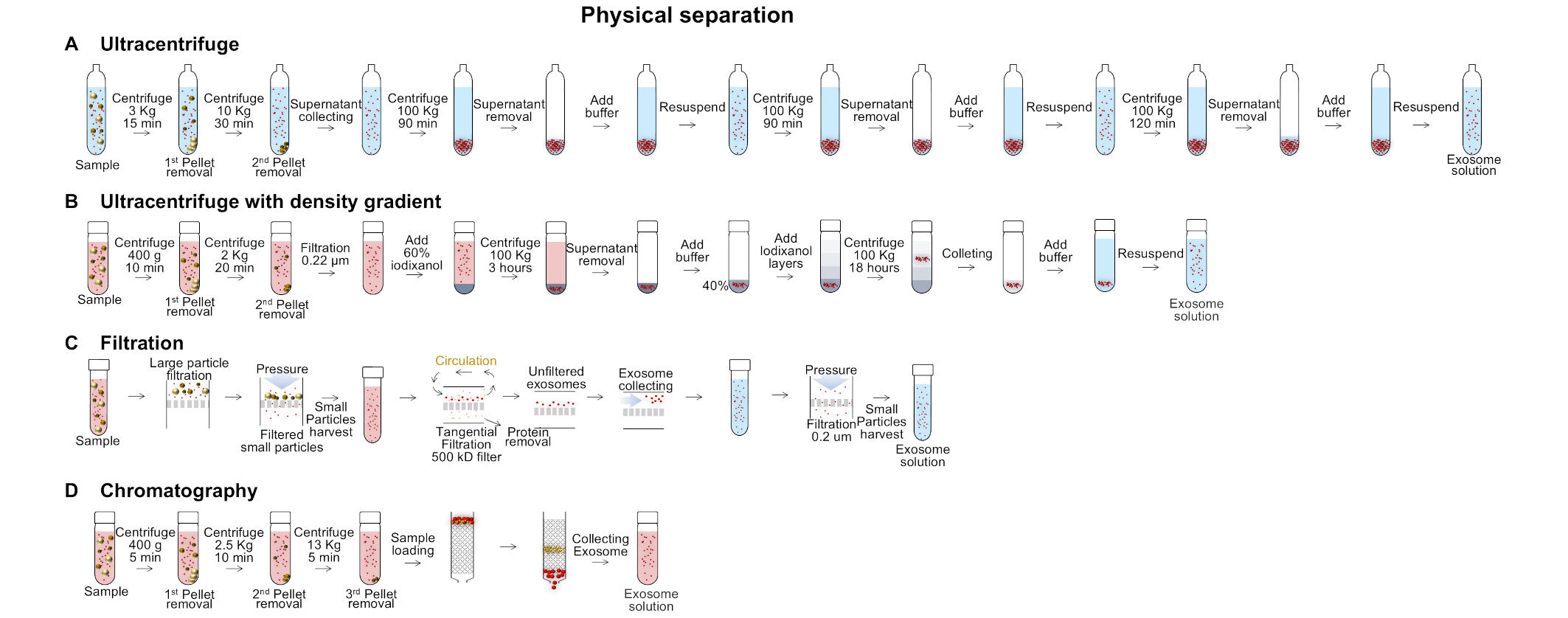


Supplementary Fig. 1| Detailed operation steps of physical isolation-based exosome separation techniques. (A) Ultracentrifugation isolates exosomes using strong centrifugal forces generated at a high rotational speed. (B) Density gradient centrifugation using a buffer consisting of a density gradient so that the exosomes stop at the same point as the density of the exosomes. (C) Filtration is achieved by applying pressure to the sample through a filter with a pore size similar to that of exosomes. (D) Size exclusion chromatography utilizes the different mobility of exosomes according to particle size through a porous structure.


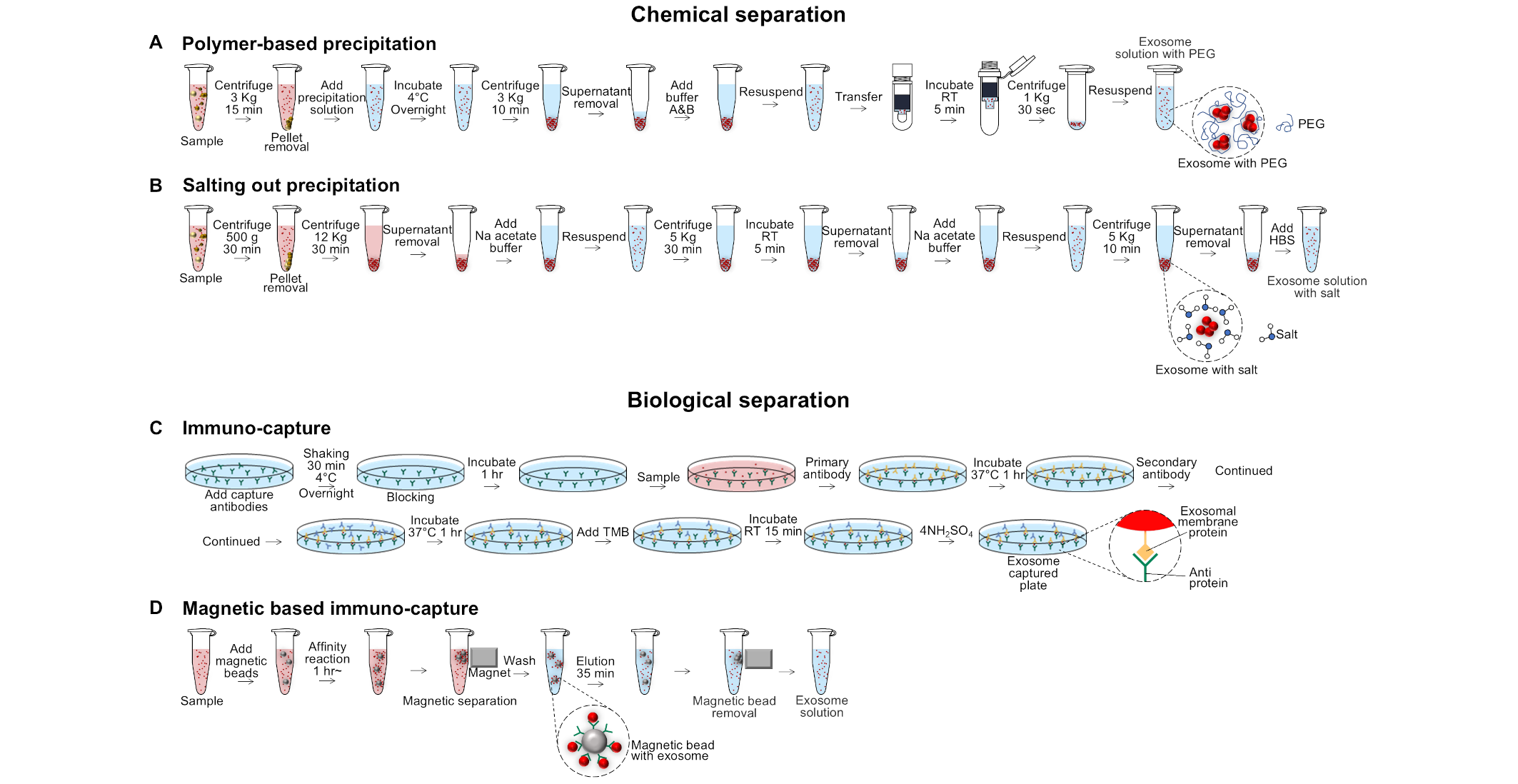


Supplementary Fig. 2| Detailed operation steps of chemical precipitation, immuno-capture based exosome separation techniques. (A) Precipitation that aggregates exosomes by forming a network with polyethylene glycols (PEG). (B) Salting out that precipitates exosomes by adding acetate to the buffer to adjust the pH. (C) Immuno-capture is based on the binding reaction between exosome membrane proteins and anti-membrane proteins. (D) Magnetic immuno-capture that attaches antibodies to magnetic beads to hold exosomes through an immune reaction and separate them by using a magnetic field.


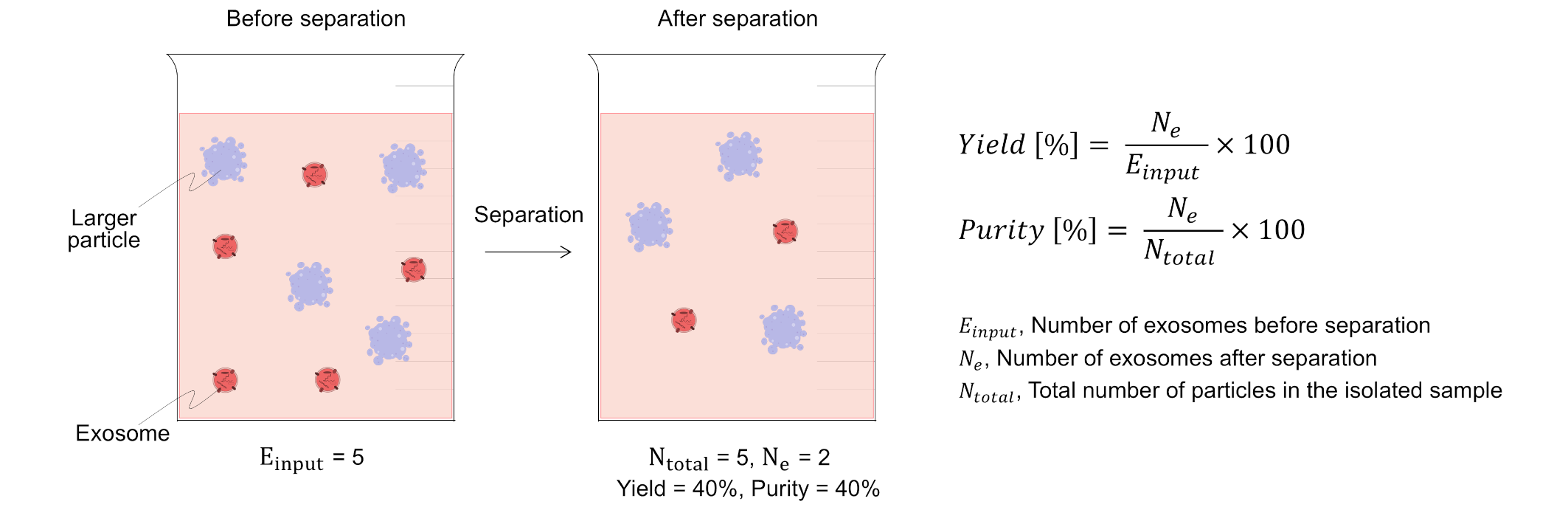


**Supplementary Fig. 3** **Quantitative definitions of exosome yield and purity.** Yield refers to the proportion of exosomes recovered after separation relative to the total number of exosomes originally present in the sample. $Yield \left[ \% \right]=\frac{N_{e}}{E_{input}}\times100$; $E_{input}$, Number of exosomes before separation; $N_{e}$, Number of exosomes after separation. Purity refers to the proportion of exosomes in the final isolated sample. The higher the proportion of exosomes compared to other particles (e.g., microvesicles, apoptotic bodies, cell debris), the greater the purity.$Purity \left[ \% \right]=\frac{N_{e}}{N_{total}}\times100$; $N_{total}$, Total number of particles in the isolated sample; $N_{e}$, Number of exosomes after separation.
